# Devices measuring transepidermal water loss: A systematic review of measurement properties

**DOI:** 10.1111/srt.13159

**Published:** 2022-04-12

**Authors:** Tanja Klotz, Abdullah Ibrahim, Guy Maddern, Yugesh Caplash, Marcus Wagstaff

**Affiliations:** ^1^ Adelaide Medical School The University of Adelaide Adelaide Australia; ^2^ Department of Occupational Therapy Royal Adelaide Hospital Adelaide Australia; ^3^ Department of Plastic and Reconstructive Surgery Royal Adelaide Hospital Adelaide Australia; ^4^ Discipline of Surgery University of Adelaide The Queen Elizabeth Hospital Adelaide Australia

**Keywords:** device, instrumentation, skin, systematic review, transepidermal water loss (TEWL), water loss (insensible)

## Abstract

**Objective:**

The objective of this review is to examine the reliability and measurement error of devices that measure transepidermal water loss (TEWL).

**Introduction:**

TEWL is a physiological property of skin which increases when the epidermis is damaged. It is, therefore, a commonly utilised measure of skin barrier integrity. Devices measuring TEWL are available as open, semi‐open or closed chamber. Studies of reliability examine the consistency of measurement, and/or responsiveness whereas measurement error scores in absolute terms the amount of error due to sources of variation.

**Inclusion criteria:**

Studies examining the reliability and/or measurement error of TEWL measurement devices were included. Studies that only report on measurement of TEWL outcomes without examination of reliability and/or measurement error were excluded.

**Methods:**

The search strategy aimed to locate published and unpublished studies. Databases searched included PubMed, Embase, CINAHL and Web of Science, utilising identified keywords and limited to studies in English. Grey literature sources were searched to identify any unpublished documents. Study selection using the inclusion criteria was then assessed by two reviewers for methodological quality utilising the COnsensus‐based Standards for the selection of health Measurement INstruments (COSMIN) risk of bias tool to assess the reliability and measurement error of outcome measurement instruments.

**Results:**

A total of 22 devices were examined in the 38 included studies. The quality of study design was on average rated as ‘Adequate’ however reliability and measurement error statistical methods were on average rated as ‘Doubtful’.

**Discussion and conclusion:**

TEWL measurement devices were found to demonstrate good reliability and frequently correlated with other devices. However, measurement error was highly variable but improves under in vitro conditions. Future research should consider risk of bias factors when designing studies.

## 1 INTRODUCTION

### 1.1 Background

Healthy skin protects the underlying structures from external factors and pathogenic microorganisms.^1^ The main chemo‐physical barrier is the stratum corneum which is the outermost layer of skin consisting of corneocytes surrounded by lipids.^2^ A decrease in the integrity of this barrier results in skin lesions that accompany many dermatological diseases.^1^ The water content of the cells in stratum corneum is 10%–15% compared to the keratinocytes at the basal area of the epidermis, consisting of 75%–85% water.^2^ The gradient of water concentration results in water loss that is higher than sweating at room temperature.^2^ This normal occurrence of insensible slow water loss of the skin is termed transepidermal water loss (TEWL), which is defined as ‘the flux density of water, which diffuses from the dermis and epidermis through the stratum corneum to the skin surface’ (p. 1050).^3^

For normal skin, under ambient conditions, TEWL oscillates between 4 and 10 g/h/m^2^.^1,2^ This water loss accounts for a total of about 500 ml per day but may increase up to 30 times higher when the epidermis is damaged.^1^, ^2^ Therefore, TEWL correlates with skin barrier function and can be a measure of dysfunction. TEWL is regarded as an important parameter when measuring skin barrier integrity. It has been highlighted as a standard measurement in guidelines and is utilised in a variety of dermatological and skin research studies.^4^, ^5^ TEWL cannot be directly observed but can be measured with devices which contain highly evolved humidity and temperature sensors measuring the density gradient of water evaporation from the skin. They are also connected to technologically advanced analysis software to provide maximum quality data output. TEWL measurement devices are available as open, semi‐open or closed chamber systems; however, it is unclear which are most valid and reliable.

The EEMCO Group, the European Group on Efficacy Measurement and Evaluation of Cosmetics and other Products, has recently (2018) published revised guidelines for in vivo measurement of water in the skin.^4^ This article reviews the measurement of hydration and TEWL.^4^ A section on TEWL measurement examines the open, semi‐open and closed chamber methods. The open chamber method includes the original Evaporimeter EP1 (Servomed AB, Stockholm, Sweden) developed in the 1980s.^4^ The practical limitations of this system are many. Measurements are prone to variability with temperature, humidity and air movement conditions.^4^ The semi‐open chamber method assists with protection from ambient air flow but does not allow for the humidity to build which can occur in a closed chamber system. The semi‐open chamber device example in the EEMCO guideline is the DermaLab (Cortex Technology, Hadsund, Denmark).^4^ An example of a closed system device is the Vapometer (Delphin Technologies Ltd, Kuopio, Finland) which has the practical advantage of being a portable hand held device, but concerns have been expressed regarding its sensitivity.^4^ The AquaFlux AF200 (Biox Systems Ltd, London, UK) and the H4500 (Nikkiso‐Therm Co. Ltd, Musashino, Japan) are additional closed chamber devices discussed in the EEMCO guideline.^4^ The authors concluded that measurements taken from different instruments cannot be compared but they are useful on their own in comparative studies, for example comparison of two moisturisers, young versus old skin, diseased versus healthy skin.

A systematic review that discusses TEWL was conducted by Lee et al.^6^ on objective scar measures. This incorporated a small section on measurement of TEWL, however, they referred to only three studies^7^, ^8^, ^9^ related to devices that measure TEWL and one study^10^ as an example of utilising TEWL as a measurement of scar outcome. The study by Fluhr et al.^7^ examines the measurements of TEWL across a wide range of disruptions to skin barrier function with a variety of methods using seven different, open and closed, devices. These were compared in vivo in humans and hairless mice. They found that the DermaLab and H4300 (Nikkiso‐Therm Co. Ltd) were less sensitive to differences in very high or very low disruptions to skin barrier function but concluded that all devices tested are reliable for measuring variations in TEWL caused by changes to skin barrier function.^7^ A systematic review was conducted by Akdeniz et al.^11^ to update reference values for healthy skin in adults. This indicated that the most common used devices in this type of research was the Evaporimeter, followed by the Tewameter. For closed chamber devices the most measurements were available for the Vapometer.^11^

Studies which examine and report on measurement devices may conclude the device to be valid, that is that they measure what it is they are supposed to measure by comparison with an accepted standard.^12^ Studies of reliability examine the consistency of measurement, and/or responsiveness whereas measurement error scores in absolute terms the amount of error due to sources of variation.^13^ Mokkink et al.^13^ who developed the risk of bias tool did so by completing a Delphi study whereby the consensus was that the intra‐class correlation coefficient (ICC) is the preferred statistical method to measure reliability. For measurement error the standard error of measurement (SEM), smallest detectable change (SDC), limits of agreement (LoA) or coefficient of variation (CV) are the preferred statistical methods.^13^

This systematic review follows the published a priori research protocol detailing the criteria upon which studies were included for appraisal, the algorithms for database searching and the method for assessment of risk of bias.^14^

### 1.2 Objectives

The aim of this systematic review was to identify all available studies examining the reliability and measurement error of different TEWL measurement devices and critically appraise these studies. The key terms for this review included skin, TEWL, water loss (insensible), instrumentation, equipment, supplies and device. The objective of this review was to evaluate the reliability and measurement error of various measurement devices in measuring skin TEWL and discuss considerations for the various TEWL measurement devices in future research.

The question of this review is: what is the statistical reliability and measurement error of published instruments that are used to measure skin TEWL?

## 2 INCLUSION CRITERIA

The inclusion criteria were guided by COnsensus‐based Standards for the selection of health Measurement INstruments (COSMIN)^15^ and the Joanna Briggs Institute (JBI) guidelines for systematic reviews.^12^

### 2.1 Population

This review considered full text studies that include observations on the measurement of TEWL by one device, comparison to another TEWL measurement device and comparison to utilisation of that same device under different conditions in an effort to report on measurement error and reliability. The review contains studies that report on skin or skin models in in vivo or in vitro conditions. Human or animal skin, intact or compromised, was appropriate for inclusion.

There were no age limits for human subjects provided the samples used were consistent or any variations were clearly specified. The operators of the device were researchers; a number of clinicians examining inter‐ and intra‐rater reliability; or the participants where self‐measurement of TEWL occurred.

### 2.2 Instruments

TEWL devices to be included were classified as open, semi‐open or closed/unventilated chamber devices. Open chamber devices consist of a hollow cylinder containing the humidity sensors that is placed in contact with the skin and open to the surrounding atmosphere at the other end.^16^ The advantage is that they do not occlude the skin and leave the microclimate of the skin relatively undisturbed, however, they are more vulnerable to environmental disturbances such as external air movements.^17^ The semi‐open chamber method assists with protection from ambient air flow but does not allow for the humidity to build which can occur in a closed chamber system. Closed chamber devices consist of a chamber containing the humidity sensors with a closed upper end which protects from external air movements. As humidity builds inside the chamber it needs to be lifted from the skin after each reading and so cannot be used for continuous TEWL measurement.^17^

### 2.3 Construct

The construct examined in this review was TEWL. TEWL is a measure of the change or flux in water vapour density at the skin surface compared to a point further away from the skin.^16^ As water is lost from the stratum corneum of the skin the humidity next to the skin surface rises above ambient humidity. Water vapour measurements are taken over an area of skin over time and the units for TEWL is stated as grams per square meter per hour or g/m^2^/h.^17^

### 2.4 Outcomes (measurement properties)

This review considered studies that included results reported as reliability and measurement error of TEWL measurement devices. The definition of measurement properties will be based on the COSMIN guidelines which requires an assessment of the quality of the studies by extracting the reliability and/or measurement error calculations.^13^, ^18^

Reliability of TEWL devices would indicate that a device consistently measures TEWL. Reliability may be reported as inter‐ and/or intra‐rater reliability. Inter‐rater reliability indicates the consistency of measures between different raters of the same sample. Intra‐rater reliability would indicate the consistency of one rater when making repeated measures on the same sample. TEWL devices produce continuous scores (as opposed to ordinal or dichotomous scores) therefore the statistics utilised to indicate reliability ought to include intra‐class correlation coefficients (ICC), Pearson correlation coefficient or Spearman correlation coefficient according to the COSMIN risk of bias tool.^13^

Measurement error is defined as the ‘systematic and random error of a patient's score that is not attributed to true changes in the construct to be measured. It refers to how close the scores of repeated measurements in stable patients are’ (Mokkink et al.,^13^ p. 8 of User Manual section). The preferred statistics used to calculate the measurement error include SEM, LoA or CV for continuous scores.^13^

Information on the sensitivity of the devices was also extracted from studies, such as, the sensitivity and performance of the device to detect changes at high or low levels of TEWL. Studies report on measurements from only one measurement device but compare its results across a range of TEWL environments such as those created by the researcher by application of irritants (e.g. sodium lauryl sulphate) or physical damage to the skin (e.g. tape stripping) to impede skin barrier function were for inclusion and their conditions noted. In addition, patient factors such as the presence of a scar or wound as a comparator to normal skin, but using two different devices on the same location were recorded.

Responsiveness factors reported in studies, for example, changes in temperature, relative humidity, angle of application of the device, length of time to take the measurement, or force of application of the device were included. Responsiveness measures reported as either correlations or differences in the change of scores were reported in the same units as TEWL, that is g/m^2^/h.

### 2.5 Types of studies

This review considered quantitative, cross‐sectional and observational studies that compare one device to another. Any quantitative comparative study design may be included however those that focus on the development and/or validation of TEWL measurement devices were prioritised. Only studies published in English were able to be included as resources were not available to translate. Studies published from database inception to the present were included when conducting searches.

### 2.6 Exclusion criteria

Studies that only use TEWL measurement devices as an outcome measure were excluded. Those that report on TEWL measurements of various skin conditions and do not report on the reliability or measurement error of the device were excluded. Components of a study that are reporting only on the values displayed by the device were not included in the synthesis of data for this review. Studies that duplicate validation data of an instrument in a previous study and do not present new measurement property data, were excluded.

## 3 METHODS

The proposed systematic review was guided by the JBI methodology for systematic reviews of measurement properties and COSMIN.^12^, ^15^ This review was submitted with PROSPERO, registration number CRD42020188586.

### 3.1 Search strategy

In an effort to maximise all available studies and ensure a comprehensive systematic review the search strategy aimed to locate both published and unpublished studies. The text words contained in the titles and abstracts of relevant articles, and the index terms used to describe the articles were used to develop a full search strategy for PubMed, Embase, CINAHL and Web of Science databases. Databases were searched from date of inception until present to allow full capture of relevant studies. Language was restricted to English as mentioned above in the inclusion criteria. Examples of search algorithms are reported in Appendix 1. It was decided to not incorporate the COSMIN sensitive search filter as the number of results was not excessive in the testing of the search strategy without it.

A search of the grey literature was also be performed to identify any unpublished documents, such as technical or research reports, doctoral dissertations, theses and conference papers.

Websites searched included:

- https://clinicaltrials.gov/—a registry and results database of publicly and privately supported clinical studies of human participants.
- www.anzctr.org.au—Australian and New Zealand Clinical Trials Registry—an online registry of clinical trials being undertaken in Australia, New Zealand and elsewhere.
- www.isrctn.com—a primary clinical trial registry recognised by the World Health Organization and International Committee of Medical Journal Editors that accepts all clinical research studies (whether proposed, ongoing or completed), providing content validation and curation and the unique identification number necessary for publication.
- www.opengrey.eu—the System for Information on Grey Literature in Europe is an open access reference on grey literature produced in Europe.
- https://www.cochranelibrary.com/central—the Cochrane Central Register of Controlled Trials.
- www.proquest.com—ProQuest Dissertations and Theses Global—a collection of dissertations and theses from around the world.
- www.worldcat.org—a network of library content and services.

### 3.2 Study selection

Following the search, all identified citations were collated and uploaded into EndNote X9.3.1, 2018 (Clarivate Analytics, PA, USA) and duplicates removed. Titles and abstracts were then screened by two reviewers independently for assessment against the inclusion criteria for the review.

The full text of selected citations was assessed in detail against the inclusion criteria by two independent reviewers. Reasons for exclusion of full text studies that do not meet the inclusion criteria were recorded and reported in the systematic review. Any disagreements that arose between the reviewers at each stage of the study selection process were resolved through discussion, a third reviewer was not required.

### 3.3 Assessment of methodological quality

JBI checklists specific to critical appraisal of the psychometric properties of measurement devices are not available. Despite there being several critical appraisal tools developed for the appraisal of studies of measurement properties, the COSMIN remains the benchmark in this area.^19^ However, the COSMIN tools have been developed specifically for patient reported outcome measures (PROMs). JBI recommends the COSMIN risk of bias tool for studies on reliability or measurement error of outcome measurement instruments recently published, which was a better fit for this systematic review topic.^12^, ^13^

Six design requirements were scored on a four‐point rating scale outlined in Table 4 of Mokkink et al.,^13^ rated as very good, adequate, doubtful or inadequate quality with an additional ‘NA’ category if required. An additional three questions on statistical methods for reliability, or two questions for measurement error, are similarly rated, however, as TEWL measurement devices produce continuous scores only questions 7a and 7b were utilised. The risk of bias tool is presented in Appendix 2.^13^ Eligible studies were appraised by two independent reviewers for methodological and outcome quality. A third reviewer was not required to resolve any disagreements.

### 3.4 Data extraction

Data were extracted from studies included in the review by the main author and reviewed by a co‐author (Abdullah Ibrahim). In addition to extraction of the statistical results reported by the included studies, information of type and name of device(s) the study has assessed, including specific information on how the device was used and from what the TEWL measurement was taken, was extracted. In addition to the data on the measurement properties of the TEWL measurement devices, where possible data in relation to the feasibility and interpretability of the instrument(s) were extracted.

## 4 RESULTS

Searches were conducted at the end of March 2021 utilising the search strategies outlined in Appendix 1 resulting in a total of 2293 studies. After removal of duplicates 838 studies remained and were screened by two of the authors (Tanja Klotz and Abdullah Ibrahim). Assessment for eligibility by both authors against the inclusion criteria identified 45 articles to partake in full text review. Seven studies were then excluded, see Appendix 3. What remained were 38 studies for inclusion, for full details see Appendix 4.

**FIGURE 1:**
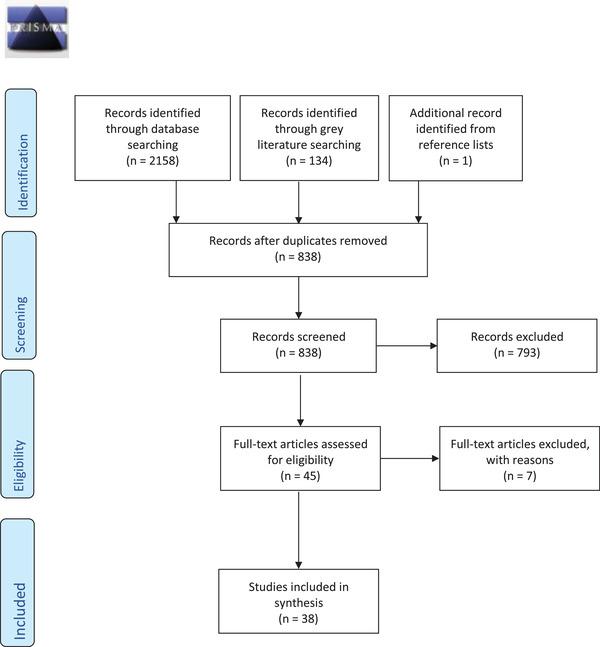
Preferred Reporting Items for Systematic Reviews and Meta‐analyses (PRISMA) 2009 flow diagram^20^

The results of this search are presented in a Preferred Reporting Items for Systematic Reviews and Meta‐analyses (PRISMA) flow diagram—Figure 1.^20^ Critical appraisal utilising the COSMIN risk of bias tool allowed for analysis of the quality of the studies with questions 1–6 and quality of statistical methods to report on reliability (question 7a) and agreement (question 7b).^13^ The results of the risk of bias assessment as agreed upon by both raters (Tanja Klotz and Abdullah Ibrahim) are presented in Table 1. Within the 38 included studies, 22 devices were reported upon. Tables 2 and 3 summarise the number of studies and devices that were included in this systematic review.

**TABLE 1:** Results of risk of bias assessment of included studies

Author	Q1	Q2	Q3	Q4	Q5	Q6	Q7a (reliability)	Q7b (agreement)
Anthonissen et al. 2013	VG	VG	VG	D	D	VG	VG	A
Barel and Clarys 1995	A	D	VG	D	D	VG	A	A
Blichmann and Serup 1987	VG	VG	VG	D	D	VG	IA	VG
Cointereau‐Chardon et al. 2020	A	VG	D	D	D	D	NA	IA
De Paepe et al. 2005	VG	VG	VG	D	D	VG	A	A
Elkeeb et al. 2010	VG	VG	VG	D	D	VG	A	NA
Farahmand et al. 2009	VG	VG	VG	D	D	D	A	A
Fell et al. 2016	VG	VG	A	A	D	VG	VG	VG
Fluhr et al. 2006	VG	VG	VG	D	D	VG	A	A
Gardien et al. 2016	VG	VG	VG	D	D	VG	VG	VG
Grinich et al. 2019	VG	D	VG	D	D	D	VG	NA
Grinich et al. 2021	VG	VG	VG	D	D	VG	VG	IA
Grove et al. 1999	A	VG	VG	D	D	VG	IA	A
Hon et al. 2018	VG	D	VG	IA	IA	D	D	NA
Hua et al. 2017	VG	D	VG	IA	IA	VG	D	D
Imhof et al. 2005	VG	VG	VG	D	D	VG	D	D
Kikuchi et al. 2017	VG	D	VG	D	D	D	VG	NA
Lau‐Gillard et al. 2010	D	D	VG	IA	IA	D	NA	D
Logger et al. 2020	VG	VG	VG	IA	IA	D	A	NA
Miteva et al. 2006	VG	VG	VG	D	D	VG	NA	IA
Murphrey et al. 2020	VG	D	VG	IA	IA	VG	VG	IA
Norlén et al. 1999	VG	VG	VG	D	D	D	NA	IA
Nuutinen et al. 2003	D	D	D	D	D	IA	D	A
Park and Tamura 1992	VG	VG	VG	D	D	D	D	NA
Pinnagoda et al. 1989	VG	VG	VG	D	D	VG	NA	IA
Rogiers 1995	VG	D	VG	D	D	VG	NA	A
Rosado et al. 2005	VG	VG	A	D	D	D	NA	D
Scott et al. 1982	VG	VG	VG	D	D	VG	NA	A
Shah et al. 2005	VG	VG	VG	D	D	VG	IA	D
Sim et al. 2019	VG	VG	A	D	D	IA	NA	IA
Smallwood and Thomas 1985	D	D	D	D	D	D	NA	IA
Steiner et al. 2011	VG	D	VG	D	D	D	A	VG
Tagami et al. 2002	VG	VG	VG	D	D	D	IA	D
Van Sam et al. 1994	VG	VG	VG	IA	IA	VG	IA	NA
Yamamura et al. 1990	VG	VG	VG	D	D	D	IA	NA
Ye et al. 2019	A	D	D	IA	IA	D	IA	D
Yoshihara et al. 2007	D	D	VG	D	D	D	NA	IA
Zhai et al. 2007	VG	D	VG	D	D	D	IA	VG

*Note*: Questions 1–7b are provided in Appendix 2.

Abbreviations: A, adequate; D, doubtful; IA, inadequate; NA, not applicable, that is the study did not report on this measurement property; VG, very good.

**TABLE 2:** Overview of studies and devices included in this systematic review

Number of included studies	38 (28 in vivo, 5 in vitro, 5 both)
Devices reported upon	22
Total number of participants (in vivo studies only)	1029 (range 1–200 per study)
Publication year, range	1982—2021

**TABLE 3:** Number of studies that refer to device brands and the properties of these devices included in this systematic review

Device	Device properties	Number of studies
Tewameters (Courage and Khazaka, Cologne, Germany)	Open chamber. Height 2 cm, diameter 1 cm. Water vapour pressure gradient is indirectly measured by two pairs of a combined thermistor and hygrosensor, present at two different heights inside a hollow cylinder.	18 (includes TM300: 10 studies)
Evaporimeter (Servomed AB, Stockholm, Sweden)	Open chamber system, diameter of 12 mm. Two pairs of sensors measuring temperature and relative humidity are placed centrally in the probe chamber.	11 (includes EP1: 9 studies)
Vapometer (Delphin Technologies, Kuopio, Finland)	Closed chamber, hand held, 1.0 cm diameter. Containing a Honeywell humidity sensor HIH 3605‐B. Single point TEWL reading.	11
AquaFlux AF200 (Biox Systems Ltd, London, UK)	Condenser‐chamber (closed) measures water vapour flux density. Upper end is closed with a condenser cooled to ‐7.65°C, acts as a sink for water vapour. Can take measurements continuously as the vapour entering the chamber is continuously removed by the condenser. Probe opening is 7 × 7 mm.	7
GPSkin (GPower, Seoul, South Korea)	Semi‐closed chamber model is similar to a closed chamber system but provides a degree of chamber ventilation. Can measure TEWL, hydration and temperature. Probe opening 11 × 14 mm. Data sent to Smartphone via Bluetooth.	7
DermaLab (Cortex Technologies, Hadsund, Denmark)	Semi‐open chamber. 1.0 cm diameter probe, software displays TEWL over time as well as single value based on average of individual recordings.	6
H4300 and H4500 (Nikkiso‐Therm Co Ltd, Musashino, Japan)	Closed chamber system, includes thermo‐ and humidity sensors. Displays evaporative quantity, relative humidity and probe temperature. No longer manufactured.	3
Noevir‐EVA	Cobalt chloride absorbed filter paper previously placed on the skin, intensity of reflected light from an LED is digitalised.	1
Norlan et al. Evaporimeter system	System that consists of evaporation unit with mounting system for sample, evaporimeter with thermistors and capacitive sensors and measurement box.	1
MEECO (MEECO, Warrington, PA, USA)	Closed loop system, results presented in p.p.m. of water loss/0.5 m^2^ h. Filled with dry nitrogen gas. Must be pushed firmly on the skin.	1
Truncated hollow cone	Closed chamber, includes measurement of skin conductance and hardness. Entrance hole diameter of 3 mm.	1
TEWL analyser CC01	Closed chamber device with crystal oscillator and shutter device which opens when pressed on the skin.	1
Smallwood and Thomas Device	An air pump draws air through the mixing chamber past humidity sensor and temperature measuring thermistor.	1

Abbreviations: LED, light emitting diode; TEWL, transepidermal water loss.

### 4.1 Quality of study design

For questions 1–6 which examined quality of the study design, on average, all studies scored ‘Adequate’. Most studies scored a ‘Very Good’ rating for questions 1 and 3 (Appendix 2) indicating that patients were stable across measurements and the conditions were similar. This was frequently indicated by the included studies reporting on rapid reassessment of subjects and stable temperature and humidity conditions. Questions 2 and 6 were on average rated as ‘Adequate’ as studies had an appropriate interval between TEWL measurements and it was identified there were additional study design flaws that had not been covered by questions 1–5. The included studies performed poorly with regards to questions 4 and 5. All of the included studies were not able to report that the assessors administering the measurement or assigning the scores did not have knowledge of the values of repeated measures on the subjects.

### 4.2 Reliability and measurement error

**FIGURE 2:**
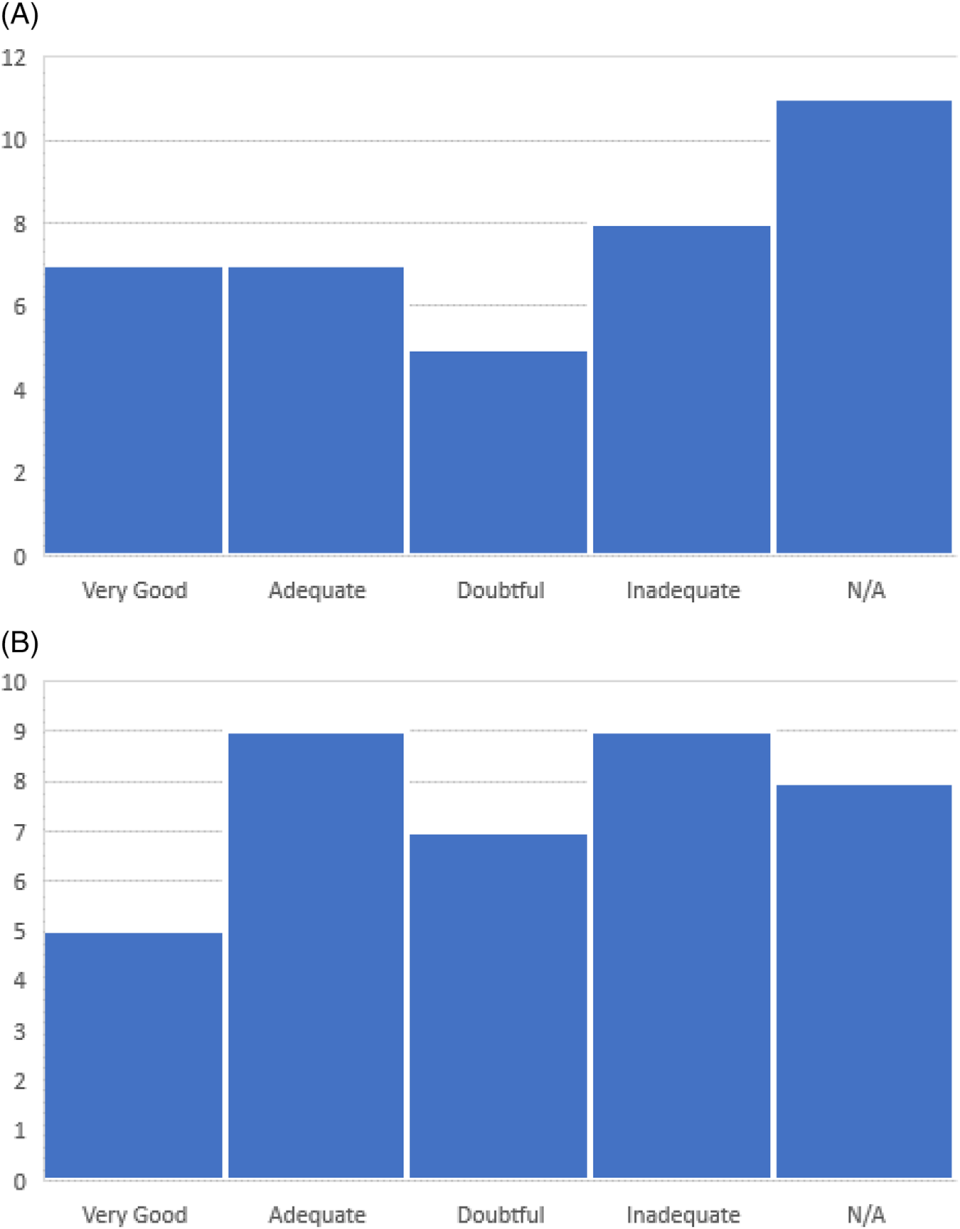
(A) Reliability ratings for all studies as per question 7a (reliability) in risk of bias assessment tool.^13^ (B) Reliability ratings for all studies as per question 7b (measurement error) in risk of bias assessment tool^13^

Of the 38 included studies, 27 contained statistics on reliability and 30 contained analysis of measurement error. An overall rating of the reliability statistics utilised were ‘Doubtful’ (median = 2.5, where doubtful has a value of 2 and adequate has a value of 3). The median value for measurement error is also ‘Doubtful’ (median = 2.0). Figure 2A,B summarises the ratings which were allocated according questions 7a and 7b of the risk of bias tool^13^ (Appendix 2).

### 4.3 Synthesis of data per device

To enable synthesis of the data in a clinically useful format the data have been pooled into Tables 4 and 5. Table 4 pools the data on reliability for each device, and Table 5, the data on agreement. An overall rating of the measurement property was obtained by allocating scores to the ratings and calculating an average over the numbers of studies assessed, that is ‘Inadequate’ = 1, ‘Doubtful’ = 2, ‘Adequate’ = 3 and ‘Very Good’ = 4. The same scoring system was applied to questions 1–6 on study design, the maximum possible score is 24, the total score is provided with a percentage. The number of participants and studies indicates the scale of the results when comparing across studies.

**TABLE 4:** Pooling of results for each device for the measurement property—reliability

Device	Number of subjects	Number of studies and reference number/s	Pooled summary	Overall rating of reliability^a^	Quality of the study design^b^
AquaFlux AF200	180	7^30^, ^31^, ^33^, ^45^, ^46^, ^47^, ^48^	Baseline TEWL measured with AquaFlux at zero time is correlated with the flux rate of tritiated water (*p* = 0.04, *r* ^2^ = 0.34). Pearson's correlation coefficient is 0.58 for AquaFlux and Vapometer and 0.88 for Tewameter and AquaFlux on human forearms. AquaFlux showed ‘moderate’ test–retest reliability (ICC = 0.58, 95% CI: 0.36‐‐0.73) which improved to ‘good’ reliability (ICC = 0.86, 95% CI: 0.76‐‐0.92) in the second trial (increased participant education). GPSkin and AquaFlux were moderately correlated by Spearman's for TEWL (*r* ^2^ = 0.48, *p* = 0.0004) when AquaFlux versus participant taken measures with GPSkin. Participant GPSkin after education TEWL measurements and the AquaFlux were moderately correlated (*r*s = 0.40, *p* = 0.0045) while investigator GPSkin TEWL measurements and the AquaFlux were weakly correlated (*r*s = 0.34, *p* = 0.0147). AquaFlux versus Vapometer *R* = 0.82 (in vivo). GPSkin versus AquaFlux: linear regression *R* = 0.8718, *p* < 0.0001, Spearman correlation coefficient *R* = 0.9256, *p *< 0.0001. In the combined cohort ICC = 0.984 (95% CI, 0.973–0.991) for AquaFlux. For subjects with ichthyosis, AquaFlux ICC = 0.976 (95% CI, 0.954–0.988), and for controls AquaFlux ICC = 0.816 (95% CI, 0.646–0.912), AquaFlux versus GPSkin for the entire cohort (*r*s = 0.743, *p* < 0.001) and moderately well correlated when analysed separately for ichthyosis (*r*s = 0.518, *p* = 0.003) and controls (*r*s = 0.536, *p* = 0.006). When the entire population was divided at the median of GPSkin measurements, the correlation between GPSkin and AquaFlux was better at the higher range (above the median, 9.7 g/m^2^/h; *r*s = 0.675, *p* < 0.001) than below the median (*r*s = 0.499, *p* = 0.008). GPSkin versus AquaFlux correlation for patient measured: *r* ^2^ (non‐lesional) = 0.72, *r* ^2^ (lesional) = 0.68 and clinician measured *r* ^2^ (non‐lesional) = 0.80, *r* ^2^ (lesional) = 0.66. Test–retest reliability ICC values for The AquaFlux was 0.90 (0.84–0.94) for non‐lesional skin and 0.76 (0.61–0.86) for lesional skin.	Adequate	18 (75%)
DermaLab	114	6^7^, ^23^, ^38^, ^51^, ^52^, ^53^	ICC ≥ 0.86 for intra‐observer reliability and ICC ≥ 0.78 for inter‐observer reliability. Tewameter versus DermaLab on scar: ICC = 0.81, *r* = 0.93, good to excellent correlation. Healthy skin: ICC = 0.52, *r* = 0.72, moderate correlation. EP1 versus DermaLab TEWL probe in vitro: *R* ^2^ = 0.9589, in vivo *R* ^2^ = 0.8665. TM300 versus DermaLab *r* = 0.940, *p* < 0.001. H4300 versus DermaLab an excellent linear relationship *R* ^2^ = 0.92; *p* = 0.0001 was found. In vivo mouse/in vivo human DermaLab Pearson correlation with: MEECO *r* = 0.9253/0.6763, H4300 *r* = 0.9536/0.7602, Vapometer *r* = 0.9716/0.9050, TM210 *r* = 0.8361/0.8639, TM300 *r* = 0.9879/0.9371, EP1 *r* = 0.9847/8735. In vivo human model correlation with temperature, *r* = 0.4184.	Doubtful	18.8 (78%)
Evaporimeter 2100	16	1^55^	Correlation with increase in air temperature *r* = 0.89. Correlation with Tewameter at different skin sites under various conditions *r* = 0.97.	Adequate	17 (70.8%)
GPSkin Barrier Light	230	2^28^, ^48^	Excellent test–retest reliability, with ICC = 0.974 (95% CI, 0.959–0.984). When the population was analysed after sub‐dividing into ichthyosis and controls, the ICCs remained excellent for subjects with ichthyosis GPSkin ICC = 0.974 (95% CI, 0.953–0.987), and was good for controls GPSkin ICC = 0.868 (95% CI, 0.745–0.937). Readings from the AquaFlux versus GPSkin were strongly positively correlated for the entire cohort (*r*s = 0.743, *p* < 0.001) and moderately well correlated when analysed separately for ichthyosis (*r*s = 0.518, *p* = 0.003) and controls (*r*s = 0.536, *p* = 0.006). When the entire population was divided at the median of GPSkin measurements, the correlation between GPSkin and AquaFlux was better at the higher range (above the median, 9.7 g/m^2^/h; *r*s = 0.675, *p* < 0.001) than below the median (*r*s = 0.499, *p* = 0.008), although correlation was still moderate to strong with both devices. TM300 versus GPSkin: cheek *r* = 0.7009, *p* < 0.0001. On the forearm, *r* ^2^ = 0.6449, *p* < 0.0001. On the dorsal hand, *R* ^2^ = 0.6991, *p* < 0.0001.	Doubtful	13.5 (56.3%)
GPSkin Pro	143	3^45^, ^46^, ^47^	GPSkin demonstrated ‘poor’ test‐‐retest reliability (ICC = 0.18, 95% CI: −0.08 to 0.42) for TEWL. After education on use: test‐‐retest reliability of GPSkin TEWL measurements improved to ‘good’ for participant (ICC = 0.89, 95% CI: 0.82‐‐0.94), investigator (ICC = 0.88, 95% CI: 0.79‐‐0.93), and participant‐‐investigator (ICC = 0.88, 95% CI: 0.79‐‐0.93) comparisons. AquaFlux versus participant taken measures with GPSkin correlation (*r* ^2^ = 0.48, *p* = 0.0004) and after patient education (*r*s = 0.40, *p* = 0.0045) while investigator GPSkin TEWL measurements and the AquaFlux were weakly correlated (*r*s = 0.34, *p* = 0.0147). GPSkin versus AquaFlux: linear regression *R* = 0.8718, *p* < 0.0001, Spearman correlation coefficient *R* = 0.9256, *p* < 0.0001. GPSkin versus AquaFlux demonstrated strong correlation for patient measured: *r* ^2^ (non‐lesional) = 0.72, *r* ^2^ (lesional) = 0.68 and clinician measured *r* ^2^ (non‐lesional) = 0.80, *r* ^2^ (lesional) = 0.66. Test–retest reliability ICC values for GPSkin ranged from 0.67 to 0.90 (CIs provided in the study).	Very good	17 (72.1%)
H4300	21	2^7^, ^53^	Correlation with gravimetric values, *r* = 0.7082. In vivo mouse/in vivo human, H4300 Pearson correlation with: MEECO *r* = 0.9746/0.4700, Vapometer *r* = 0.9811/0.7029, EP1 *r* = 0.9596/0.5858, TM210 *r* = 0.8991/0.7633, DermaLab *r* = 0.9536/0.7602. Human in vivo model correlation with temperature, *r* = 0.497. The DermaLab correlated well with H4300 with an excellent linear relationship (*R* ^2^ = 0.92; *p* = 0.0001) over a wide range of TEWL values in vivo.	Doubtful	19 (79.2%)
H4500	15	1^24^	The intra‐class correlation coefficient with 95% CI of H4500 was 0.927 (0.835–0.978). All measured sites: H4500 versus Tewameter *r* = 0.945, H4500 versus Vapometer *r* = 0.910, (all *p* < 0.001). Healthy skin: H4500 versus Tewameter *r* = 0.756, H4500 versus Vapometer *r* = 0.431. Immediately after tape stripping: H4500 versus Tewameter *r* = 0.718, H4500 versus Vapometer *r* = 0.900. 24 h after tape stripping: H4500 versus Tewameter *r* = 0.811, H4500 versus Vapometer *r* = 0.843. After 24 h of SLS: H4500 versus Tewameter *r* = 0.768, H4500 versus Vapometer *r* = 0.849.	Very good	16 (66.7%)
MEECO	NA	1^7^	Correlation with gravimetric values, *r* = 0.6825. In vivo mouse/in vivo human, MEECO Pearson correlation with: H4300 *r* = 0.9746/0.4700, Vapometer *r* = 0.9560/0.7064, EP1 *r* = 0.9247/0.8383, TM210 *r* = 0.9160/0.5598, TM300 *r* = 0.9149/0.6269, DermaLab *r* = 0.9253/0.6763. Human in vivo model correlation with temperature, *r* = 0.2793.	Adequate	20 (83.3%)
Noevir‐EVA	44	1^44^	Agreement between Noevir‐EVA and Evaporimeter *r* = 0.984	Inadequate	18 (75%)
Evaporimeter EP1	109	6^7^, ^37^, ^38^, ^39^, ^43^, ^44^	Intra‐individual reproducibility (mean difference as % of initial TEWL measurements) palm of the hand varied between 6.4% and 14.5% and for the forearm varied between 11.3% and 34.1%. EP1 Pearson correlation with gravimetric values *r* = 0.8076. In vivo mouse/in vivo human, EP1 Pearson correlation with: MEECO *r* = 0.9247/0.8383, H4300 *r* = 0.9596/0.5858, Vapometer *r* = 0.9797/0.8780, TM210 *r* = 0.8544/0.7702, TM300 *r* = 0.9875/0.8365, DermaLab *r* = 0.9847/0.8735. In vivo human model correlation with temperature, *r* = 0.2861. EP1 versus DermaLab TEWL probe in vitro: *R* ^2^ = 0.9589, in vivo *R* ^2^ = 0.8665. Correlation with weight loss of filter paper, *r* = 0.98. Correlation between body weight loss and cutaneous evaporation rate, *r* = 0.91. In vivo EP1 correlated with Noevir‐EVA *r* = 0.984.	Inadequate	19.2 (80%)
Tewameter as part of Scarbase Duo	20	1^52^	Tewameter: intra‐rater reliability (scar) ICC = 0.95, SEM = 1.17, (healthy skin) ICC = 0.87, SEM = 0.74; inter‐rater reliability (scar) ICC = 0.96, SEM = 1.12, (healthy skin) ICC = 0.90, SEM = 0.75. Concurrent validity of TEWL measurements between the Tewameter and the DermaLab: scar ICC = 0.81, *r* = 0.93, good to excellent. Healthy skin: ICC = 0.52, *r* = 0.72, moderate correlation.	Very good	20 (83%)
Tewameter TM210	57	7^7^, ^9^, ^30^, ^33^, ^34^, ^35^, ^55^	Correlation with increase in air temperature *r* = 0.88. Correlation with Evaporimeter 2100 at different skin sites under various conditions *r* = 0.97. Spearman's correlation coefficients comparing Tewameter to Vapometer reported as varying from 0.503 to 0.966 (not specific for device). Baseline TEWL correlates with the flux of tritiated water (*p* = 0.00, *r* ^2^ = 0.50). Pearson's correlation coefficient is 0.70 for Vapometer and Tewameter, and 0.88 for AquaFlux and Tewameter. Correlation with gravimetric values, *r* = 0.7666. In vivo mouse/in vivo human, TM210 Pearson correlation with: MEECO *r* = 0.9160/0.5598, H4300 *r* = 0.8991/0.7633, Vapometer *r* = 0.8759/0.8926, EP1 *r* = 0.8544/0.7702, TM300 *r* = 0.8353/0.7325, DermaLab *r* = 0.8361/0.8639. Human in vivo model correlation with temperature, *r* = 0.3637. For TEWL values of forearm locations, there were no statistically significant differences between the mean values measured by TM210 and Vapometer (*p* = 0.68–0.90). For TEWL values of forehead locations, there was significant difference between the mean values of measured by TM210 and Vapometer (*p* = 0.0049–0.04). There was neither a statistically significant difference between TM210 and Vapometer after 10, nor after 20, strips.	Doubtful	18.7 (77.9%)
Tewameter TM300	390	7^7^, ^21^, ^22^, ^23^, ^24^, ^27^, ^28^	Correlation with gravimetric values, *r* = 0.7557. In vivo mouse/in vivo human, TM300 Pearson correlation with: MEECO *r* = 0.9149/0.6269, H4300 *r* = 0.9513/0.6475, Vapometer *r* = 0.9750/0.7833, EP1 *r* = 0.9875/0.8365, TM210 *r* = 0.8353/0.7325, DermaLab *r* = 0.9879/0.9371. Human in vivo model correlation with temperature, *r* = 0.4025. For burn scars: intra‐observer reliability ICC: 0.89–0.92 (excellent agreement). Inter‐observer reliability ICC: 0.85–0.94 (excellent agreement). TM300 versus Vapometer, *r* = 0.357, *p* < 0.05 on the forearms of patients with AD. TM300 (open chamber, standard) versus TM300 (closed, semi‐permeable ring) ICC is 0.98 (95% CI: 0.97–0.98) and 0.70 (95% CI: 0.65–0.74) for the Vapometer, on palm and dorsum of hands. DermaLab versus TM300, *r* = 0.940, *p* < 0.001 on the face and forearm of normal subjects. The ICC with 95% CI of TM300 was 0.93 (0.842–0.979). All measured sites: H4500 versus TM300 *r* = 0.945, Vapometer versus TM300 *r* = 0.939 (all *p* < 0.001). Healthy skin: H4500 versus TM300 *r* = 0.756, Vapometer versus TM300 *r* = 0.492. Immediately after tape stripping: H4500 versus TM300 *r* = 0.718, Vapometer versus TM300 *r* = 0.850. 24 h after tape stripping: H4500 versus TM300 *r* = 0.811, Vapometer versus TM300 *r* = 0.861. After 24 h of SLS: H4500 versus TM300 *r* = 0.768, Vapometer versus TM300 *r* = 0.533. TM300 versus GPSkin: cheek *r* = 0.7009, *p* < 0.0001. On the forearm, *r* ^2^ = 0.6449, *p* < 0.0001. On the dorsal hand, *R* ^2^ = 0.6991, *p* < 0.0001.	Adequate	16.1 (67%)
Vapometer	108	6^7^, ^22^, ^27^, ^30^, ^31^, ^32^	Vapometer shows no statistically significant correlation with the flux rate of tritiated water (*p* = 0.07, *r* ^2^ = 0.31). Correlation with gravimetric values, *r* = 0.7630. In vivo mouse/in vivo human, Vapometer Pearson correlation with: MEECO *r* = 0.9560/0.7064, H4300 *r* = 0.9811/0.7029, TM210 *r* = 0.8759/0.8926, EP1 *r* = 0.9797/0.8780, TM300 *r* = 0.9750/0.7833, DermaLab *r* = 0.9716/0.9050. Human in vivo model correlation with temperature, *r* = 0.2646. TM300 versus Vapometer correlation *r* = 0.357, *p* < 0.05. AquaFlux versus Vapometer *R* = 0.82. Evaporation rate of Vapometer was *r* = 0.99, *p* < 0.001 until the evaporation was 200 g/m^2^/h from the petri dish. The ICC comparing the TM300 open chamber with Vapometer is 0.70 (95% CI: 0.65–0.74).	Adequate	16.8 (70.1%)
Vapometer SWL‐2	37	3^9^, ^24^, ^33^	Spearman's correlation coefficients comparing Tewameter to Vapometer reported as varying from 0.503 to 0.966. Pearson's correlation coefficient is 0.58 for AquaFlux and Vapometer, 0.70 for Vapometer and Tewameter. Intra‐rater reliability: ICC with 95% CI of Vapometer was 0.856 (0.697–0.955). All measured sites: Vapometer versus Tewameter *r* = 0.939, Vapometer versus TM300 *r* = 0.939 (all *p* < 0.001). Healthy skin: H4500 versus Tewameter *r* = 0.756, Vapometer versus TM300 *r* = 0.492. Immediately after tape stripping: H4500 versus Tewameter *r* = 0.718, Vapometer versus TM300 *r* = 0.850. 24 h after tape stripping: H4500 versus Vapometer *r* = 0.843, Vapometer versus TM300 *r* = 0.861. After 24 h of SLS: H4500 versus Tewameter *r* = 0.768, Vapometer versus TM300 *r* = 0.533.	Adequate	18 (75%)
Vapometer SWL‐3	19	2^34^, ^35^	For TEWL values of all forearm locations, there were no statistically significant differences between the mean values measured by two TM210 instruments and Vapometer (*p* = 0.68–0.90). For TEWL values of forehead locations, there was significant difference between the mean values of both TM210 devices and Vapometer (*p* = 0.0049–0.04). There was no a statistically significant difference between bilateral sites in measures by TM210 and Vapometer devices, neither after 10 nor after 20 strips.	Inadequate	18 (75%)

Abbreviations: CI, confidence interval; ICC, intra‐class correlation coefficient; SEM, standard error of measurement; SLS, sodium lauryl sulphate; TEWL, transepidermal water loss.

^a^ Where more than one study was pooled, per device, an average rating was calculated from the result of the risk of bias assessment tool, question 7a.

^b^ Quality of the study design: from studies where data were obtained per device. Ratings of questions 1–6 of the risk of bias assessment tool totalled scores achieved. Maximum of 24 points, percent in brackets (%)

**TABLE 5:** Pooling of results for each device for the measurement property—measurement error

Device	Number of subjects	Number of studies and reference number/s	Pooled summary	Overall rating of reliability^a^	Quality of the study design^b^
AquaFlux AF200	87	4^31^, ^33^, ^46^, ^48^	AquaFlux CV 4.8%–31.1% (on human forearm). For AquaFlux TEWL measurements in vivo SD varied between 0.81 g/m^2^/h, CV = 6.4% at wrist and SD 0.16 g/m/h, CV = 2.0% in middle of forearm. Compared to SD 0.09 g/m/h and CV 1.2% in vitro.	Doubtful	18.5 (77.1%)
DermaLab	114	6^7^, ^23^, ^38^, ^51^, ^52^, ^53^	SEM ≤1.74 for intra‐observer reliability and SEM ≤2.76 for inter‐observer reliability. The Bland–Altman plots for agreement between DermaLab and Tewameter on scars show the bias of the mean is high, suggesting that a systematic error could be detected. The DermaLab systematically measures 2.5 g higher than the Tewameter. The CV for DermaLab in vivo was 18.0% (Fluhr et al.), 0%–20.75% (Hua et al.), 8.33%–12.5% (Tagami et al.) and in vitro varied from 4.3% to 20.7%.	Doubtful	18.8 (78%)
Evaporimeter 2100	16	1^56^	The CV varies from 3% to 6% at different anatomical sites.	Adequate	17 (70.8%)
GPSkin Barrier Light	230	2^28^, ^48^	Bland–Altman analysis was performed to assess agreement between AquaFlux and GPSkin, which was worse at higher TEWL values for GPSkin. GPSkin mean ± SEM, in the cheek (12.34 ± 0.65), forearm (10.87 ± 0.54), dorsal hand (14.96 ± 0.61).	Inadequate	13.5 (56.3%)
GPSkin Pro	70	3^46^, ^49^, ^50^	TEWL arm mean = 2.96, SD = 4.49. TEWL face: mean = 3.85, SD = 7.06. Coefficient of determination shows 0.8613, indicating a good agreement of a linear relation between GPSkin and truncated hollow cone device. GPSkin demonstrated poor agreement with AquaFlux in Bland–Altman plots. GPSkin consistently measured lower mean values for TEWL compared to the AquaFlux and was most significant at higher TEWL values.	Inadequate	17 (75%)
H4300	21	2^7^, ^53^	In vivo on human forearm CV = 12.4. H4300 had variation of ∼15% when measured across different anatomical sites (higher on cheek than other areas). H4300: cheek (6.7 ± 0.6), forearm (2.3 ± 0.2), leg (2.8 ± 0.4) (*p* < 0.0001), lesional skin (11.5 ± 7.2) (*p* = 0.0006).	Doubtful	19 (79.2%)
MEECO	NA	1^7^	In vivo on human forearm CV = 16.6.	Adequate	20 (83.3%)
Norlan Evaporimeter	36	1^56^	Precision in vitro is 11%, and in vivo is 36%.	Inadequate	18 (75%)
Evaporimeter EP1	115	6^7^, ^37^, ^38^, ^40^, ^41^, ^42^	Intra‐individual reproducibility on palm of hand: CV = 9.4%. Intra‐individual reproducibility on the forearm = 9.1%. In vivo conditions CV reported as 13.5, ranged from 6.2% to 46.8%. Blank patches CV = 45% and SLS CV = 26%. Untreated skin: mean TEWL values was 0.32, SEM ±0.02. Tape stripped skin evaporimeter mean TEWL 7.41, SEM ±0.16. In vitro: the SDs of the mean for each probe decreased from 0.8 to 0.2 g/m^2^/h after stabilisation. In vivo: SDs of the mean for each probe decreased from about 0.4 to 0.2 g/m^2^/h.	Adequate	19.4 (81%)
Smallwood and Thomas Device	2	1^57^	The monitor can measure evaporative water loss at normal rates of 10–20 g/m/ h to an accuracy of about 10% of the reading.	Inadequate	12 (50%)
Tewameter as part of Scarbase Duo	20	1^52^	Moderate SEM values (0.74–1.17) for Tewameter. The Bland–Altman plots for agreement between the Tewameter and DermaLab on scars show the bias of the mean is high, suggesting that a systematic error could be detected. The limits of agreement are far apart, suggesting that the high correlation between the two measurement methods is not supported by high agreement.	Very good	20 (83%)
Tewameter TM200	21	1^41^	For blank versus SLS patches the values for TM200 were 30.3 ± 11.6 (CV = 38%) versus 8.1 ± 1.4 (CV = 17%), respectively.	Adequate	18 (75%)
Tewameter TM210	72	7^7^, ^9^, ^26^, ^33^, ^34^, ^35^, ^55^	The range of the CV for the Tewameter varies from 6% to 13% at different anatomical sites. Smaller differences in TEWL could be detected with the Tewameter at tape stripping skin. Based on the standard errors of the differences and the mean square error from the analysis of variance, the Tewameter provided more precise results (compared to Vapometer) when measuring the effect of a moisturiser. Tewameter CV ranged from 17.3% to 60% (in vivo, human skin). CV measured as 12.8 in vivo. The CV under normal conditions averaged 6.75. For t1/2evap (evaporation half‐life) the TM210 provided higher TEWL results and slower decay and was statistically different from the TM300 (*p* = 0.003). CV for TM210 ranged from 9.2% to 25% (in vivo). Open chamber: 10 strips—CV 41.7%. 20 strips—CV 33.5%.	Adequate	18.3 (76.3%)
Tewameter TM300	336	8^7^, ^21^, ^23^, ^25^, ^26^, ^27^, ^28^, ^29^	CV for TM300 is 1.9 on normal human ventral forearms. On burn scars intra‐observer SEM: 2.38–2.68 g/m^2^/h. Inter‐observer SEM: 1.76–3.97 g/m^2^/h. Bland–Altman plots showed relatively wide LoA values for scar and healthy skin. On healthy faces the TM300 CV 0.86%–17.70%, mean = 6.08, SD = 3.57. SDs varied from 1% to 6% for the heated petri dish and 2%–9% for the unheated petri dish. The SD for the measurements completed on the calibration bottle reached a maximal of 0.4%. Under extreme conditions the TM300 performed better than the TM210 (*p* = 0.310). For t1/2evap (evaporation half‐life) the TM210 provided higher TEWL results and slower decay and was statistically different from the TM300 (*p* = 0.003). The mean difference for open (TM300 std) minus closed (TM300 semi‐permeable ring) method is 1.3 g/m^2^ h, with agreement limits of ‐5.4 and 8.2 g/m^2^ h. Comparing the Vapometer against the TM300 open chamber, the mean difference is 1.2g/m^2^ h but the agreement limits are within the range of ‐35.8 and 38.3 g/m^2^ h difference. In the cheek TM300 mean ± SEM was 19.08 ± 0.77, forearm 17.8 ± 0.73, dorsal hand 21.57 ± 0.8. In dogs results for T300: back (mean = 17.78, variance 1.26, SD 1.12), leg (mean 23.22, variance 5.87, SD 2.42), tail base (mean 26.58, variance 8.48, SD 2.91) and shoulder (mean 65.82, variance 7.81, SD 2.79).	Doubtful	16.8 (70%)
TEWL Analyser CC01	21	1^29^	For the CCO1 on dogs: back (mean 20.56, variance 1.05, SD 1.02), leg (mean 23.4, variance 1.77, SD 1.33), tail base (mean 26.76, variance 5.9, SD 2.43), shoulder (mean 40.19, mean 0.58, SD 0.76). The lower variance and SD indicate more reliable results with the CC01.	Inadequate	14 (58.3%)
Truncated hollow cone	NA	1^50^	THC sensitivity of 0.0068 (%/s)/(g/m^2^/h) with the high linearity of 99.63%. Coefficient of determination shows 0.8613, indicating a good agreement of a linear relation between THC and GPSkin in vitro.	Inadequate	16 (66.7%)
Vapometer	28	4^7^, ^27^, ^31^, ^32^	In vivo on human forearm CV = 12.8. Results from 200 measurements using an in vitro source had an average of 9.02 g/m^2^/h and a CV of 10.3%. The Vapometer in vivo source CV varied from 8.2% to 12.3%. CV = 8.0% for the forearm, 10.1% for the palm of the hand and 4.0% for the petri dish. Vapometer versus TM300 open chamber, the mean difference is 1.2g/m^2^ h but the agreement limits are within the range of ‐35.8 and 38.3 g/m^2^ h difference.	Adequate	16.8 (70.1%)
Vapometer SWL‐2	22	2^9^, ^33^	Ten consecutive measurements for each volunteer. A shift in an upward direction was observed going from 5 ± 3 g/h m^2^ (first replicate) to 7 ± 3 g/h m^2^ (10th replicate). The variability of each replicate, however, remained the same (Levene's test for equality of variances, *p* = 0.778). When the higher sets of readings were compared with the first set, a significant difference was found for the fifth and the seventh reading onwards, meaning that reproducible results were only obtained for the first four sets of readings. Vapometer CV 11.5%–49.6% (in vivo, human skin)	Adequate	19 (79.2%)
Vapometer SWL‐3	19	2^34^, ^35^	CV for the Vapometer ranged from 14.9% to 21.2% (in vivo, human skin). Inter‐individual CV after 10 strips—2.4 ± 1.4, CV = 60.5%. 20 strips—TEWL 4.6 ± 2.2, CV = 47.8%.	Adequate	18 (75%)
Vapometer SWL‐5	23	1^36^	Short‐coated dog, CV at various sites on various days ranged from 7.3% to 76.9% with a mean of 33.4% (95% CIs ranged from 29.5% to 37.2%). Long‐coated dog, CV ranged from 4.7% to 43% with a mean of 20.3% (95% CIs ranged from 18% to 22.7%). CVs were significantly lower in both axillae compared to the top of head and between shoulders, and on the right leg compared to between shoulders (Tukey's multiple comparison, *p* < 0.05).	Doubtful	11 (45.8%)

Abbreviations: CI, confidence interval; CV, coefficient of variation; LoA, limits of agreement; SD, standard deviation; SEM, standard error of measurement; SLS, sodium lauryl sulphate; TEWL, transepidermal water loss.

^a^ Where more than one study was pooled, per device, an average rating was calculated from the result of the risk of bias assessment tool, question 7b.

^b^ Quality of the study design: from studies where data were obtained per device. Ratings of questions 1–6 of the risk of bias assessment tool totalled scores achieved. Maximum of 24 points, percent in brackets (%).

The following synthesis of the data does not include those devices that could not be grouped with others from the same manufacturer or were included in only one or two studies. This included the Evaporimeter 2100 (Servomed AB), H4300, H4500, Noevir‐EVA, MEECO (MEECO, Warrington, PA, USA), Norlan Evaporimeter, and the Smallwood and Thomas Device.

#### 4.3.1 Tewameter devices

Four different Tewameter devices were examined, the TM200, TM210, TM300 and the Tewameter as part of the Scarbase Duo. The TM300 was the most extensively studied device due to its inclusion in 10 studies,^7^, ^21^, ^22^, ^23^, ^24^, ^25^, ^26^, ^27^, ^28^, ^29^ two of which had the greatest number of participants in the included studies.^22^, ^28^ The quality of the study design for assessing reliability and measurement error ranged from 16.1 to 20 out of a total of 24 points across the six questions (Tables 4 and 5) in the risk of bias tool (Appendix 2). The studies which included the TM300 scored lowest in study design compared to studies with other Tewameters.

The Tewameters correlated with many other devices. For the TM210, the correlation coefficients when tested against other devices ranged from 0.503 to 0.97 and did not correlate strongly with the flux of titrated water and temperature.^7^, ^30^ For the TM300, the correlation coefficients when tested against other devices ranged from 0.357 to 0.9879 and also did not correlate strongly with temperature.^7^ However, statistical methods used were frequently correlation coefficients and occasionally ICCs. As a result, the statistical methods to measure reliability of TM210 were doubtful but the methods improved to adequate for the TM300. ICC values indicated the Tewameter has excellent intra‐ and inter‐rater reliability. The exception being agreement between the Tewameter as part of the Scarbase Duo and DermaLab on healthy skin which demonstrated moderate agreement.

Table 5 shows the CV was often the statistical method utilised to report on measurement error. CVs varied greatly from 0.86% to 60% across the included studies on humans and dogs. Variation was less extensive for the in vitro study including the TM300, reported as a standard deviations of 1%–9%.^25^

#### 4.3.2 Vapometer devices

Four Vapometers were utilised in 11 of the included studies. Many of the studies did not specify a model,^7^, ^22^, ^27^, ^30^, ^31^, ^32^ the remainder specified the models SWL‐2,^9^, ^24^, ^33^ SWL‐3^34^, ^35^ and the SWL‐5.^36^ The quality of the study design for assessing reliability and measurement error ranged from 11 to 19 out of a total of 24 points across the six questions (Tables 4 and 5) in the risk of bias tool (Appendix 2). The study which included the SWL‐5 model scored the lowest in study design compared to other Vapometers.^36^

The Vapometer did not correlate with the flux of titrated water.^30^ When compared with Tewameters the correlation coefficients varied greatly from 0.357 to 0.9750 (see Table 4), however, when an ICC was implemented it was shown to have fair agreement (ICC = 0.70) with the Tewameter TM300 specifically.^27^ The intra‐rater reliability of the Vapometer was shown to be good (ICC = 0.856).^24^ Most studies utilised ‘Adequate’ statistical methods to report on reliability of the Vapometer devices except for the SWL‐3 model which was rated as ‘Inadequate’ across two studies^34^, ^35^ (Table 5).

CV reported in studies under in vivo conditions were from 4.7% to 76.9%. Lower measurement error was reported in in vitro conditions as reported CVs ranged from 4.0% to 10.3%. Most studies utilised ‘Adequate’ statistical methods to report on measurement error of the Vapometer devices (Table 5).

#### 4.3.3 Evaporimeter EP1

The EP1 was examined in nine of the included studies.^7^, ^37^, ^38^, ^39^, ^40^, ^41^, ^42^, ^43^, ^44^ Almost half of the studies were conducted, or has a component of, in vitro samples. The quality of the study design scored 80% overall whereas the rating of the statistics used for measurement properties was ‘Inadequate’ for the measurement of reliability (Table 4) and ‘Adequate’ for the measurement of measurement error (Table 5). The EP1 generally correlated well with the devices it was compared against with correlations coefficients ranging from 0.585 to 0.9847 under in vivo conditions and was similar for in vitro conditions. Assessment of the accuracy of the device in in vivo conditions resulted in CV values ranging from 6.2% to 46.8%.

#### 4.3.4 AquaFlux

The AquaFlux was included in seven of the included studies.^30^, ^31^, ^33^, ^45^, ^46^, ^47^, ^48^ The studies were predominantly conducted in in vivo conditions. The quality of the study design for these studies scored 69% overall, whereas the rating of the statistics used for measurement properties was ‘Adequate’ for the measurement of reliability (Table 4) and ‘Doubtful’ for the measurement of measurement error (Table 5). Four recent studies^45^, ^46^, ^47^, ^48^ included the GPSkin with the AquaFlux demonstrated they are moderately correlated. The AquaFlux showed moderate to good intra‐rater reliability with ICC values ranging from 0.58 to 0.90 across varying conditions under in vivo conditions (Table 4). Assessment of the accuracy of the device in in vivo conditions resulted in CV values ranging from 2.0% to 31.1% compared to 1.2% in in vitro conditions (Table 5).

#### 4.3.5 GPSkin

The GPSkin Pro^45^, ^46^, ^47^, ^49^, ^50^ and GPSkin Barrier Light^28^, ^48^ were examined in seven of the studies and are the most recent of all included studies, with publication year ranging from 2019 to 2021. All were conducted under in vivo conditions. The quality of the study design for the studies examining the GPSkin Pro scored 70% and the GPSkin Barrier Light scored 56.3%. The ratings of the statistics to examine the measurement property of reliability was ‘Very Good’ for those studies that included the GPSkin Pro and ‘Doubtful’ for those that included the GPSkin Barrier Light (Table 4).

The intra‐rater reliability of the GPSkin Barrier Light was rated as excellent for the combined cohort of controls and subjects with ichthyosis (ICC = 0.974, Table 5) and correlated with the AquaFlux^48^ and Tewameter TM300.^28^ GPSkin Pro rated poorly for intra‐rater reliability prior to the provision of education on its use but improved thereafter. It was weakly correlated with the AquaFlux in one study^45^ but correlated well in two other studies.^46^, ^47^ Both devices showed greater variation from other devices as higher values of TEWL were recorded. However, measurement error statistics were of lesser quality as they were rated as ‘Inadequate’ for both devices (Table 5).

#### 4.3.6 DermaLab

The DermaLab was included in six of the included studies.^7^, ^23^, ^38^, ^51^, ^52^, ^53^ The studies were predominantly conducted in in vivo conditions. The quality of the study design for these studies scored 78%, whereas the rating of the statistics to examine the measurement properties was ‘Doubtful’ for the measurement of reliability (Table 4) and measurement error (Table 5). The DermaLab demonstrated good inter‐ and intra‐rater reliability^51^ and correlated with other devices (Table 4). Measurement error of the device under in vivo conditions reported as CV ranged from 0% to 20.75%^23^ and in in vitro conditions ranged from 4.3% to 20.7%^53^ (Table 5).

## 5 DISCUSSION

There are numerous devices measuring TEWL that were examined in this systematic review. Most examined devices are commercial instruments and a few have been examined as single device projects. The quality of devices is predominantly reasonable, and their designs (e.g. open vs. closed), have positive and negative qualities. Selecting a device requires consideration of the qualities of each. Therefore, referring to a systematic review of measurement properties may be helpful to clinicians and researchers in device selection. Through the process of database searching, identification of studies and appraisal, 38 studies were identified encompassing 22 devices. The included studies span the last 40 years during which time electronics and data analysis has evolved significantly over this time. Analysis of the study design and quality of statistics used to determine reliability (including correlations) and measurement error of each instrument resulted in a narrative synthesis of the extracted data from the studies to reflect the quality of the included studies and a summary of the data on the main devices that were reported upon.

Although the Tewameter TM300 was included in the greatest number of studies this has been replaced by the manufacturers with the Tewameter TM Hex. The Evaporimeter EP1 is no longer available and the studies in this review including this device ranged from 1982 to 2006 and average 29 years ago from the current date (2021). In a systematic review of TEWL measurements in healthy adults the Evaporimeter was the most frequently used (in 82 studies) closely followed by the Tewameter (in 76 studies).^11^

Frequently the model number was not supplied in studies including the Vapometer, yet the current model, the SWL‐5, was identified in only one study from 2010.^36^ The studies with unspecified models spans 2003–2018.^7^, ^22^, ^27^, ^30^, ^31^, ^32^ The DermaLab did not have model numbers reported, however it appears the software has been updated as the screen appearance has changed. It is unknown and undocumented as to whether the software updates have changed the TEWL readings from the device. There appears to have been an influx of studies completed utilising the GPSkin devices, with seven studies published over the last 2 years.^28^, ^45^, ^46^, ^47^, ^48^, ^49^, ^50^ Further investigation uncovered that three of the studies had devices provided by the manufacturer of the device, GPower, however two of these studies were completed by the same author.^45^, ^46^, ^48^

Across all the studies the average rating for the quality of the statistics for reliability and measurement error was ‘Doubtful’. For reliability, only seven studies achieving a result of ‘Very Good’ as they utilised ICC statistics, and described the model which matched the study design. When devices were compared against each other they would generally correlate well. Inter‐rater reliability, was reported as excellent for the Tewameter and ‘Good’ for the DermaLab. However, most studies examining rater reliability assessed only intra‐rater reliability which was rated ‘Moderate’ to ‘Good’ for the Vapometer, AquaFlux and DermaLab with the Tewameter and GPSkin achieving ‘Excellent’ intra‐rater reliability.

Five of the studies which included measurement error analysis were able to be rated as ‘Very Good’ as they utilised CV, SEM or LoA which matched the study design and data. Accuracy consistently improves with in vitro conditions, as expected, as these conditions are better controlled. What many studies demonstrated was that although a high correlation between devices is achievable they are not supported by high measurement agreement. A conclusion that was also reported by Anthonissen et al.^51^

Despite the correlations noted in many studies between devices there is still no gold standard. The use of calibration methods in some studies was utilised in an attempt to demonstrate accuracy however the conditions were therefore also an in vitro model which would naturally result in higher accuracy than in vivo conditions which is predominantly how TEWL measurement devices are used clinically. No ‘gold standard’ device has yet been declared however as research and development of the devices continues accuracy should improve. Akdeniz et al.^11^ in their systematic review also found that there is no evidence to indicate one available device was superior to another.

Clinical research should increase by linking devices to mobile applications for data production and collection and will allow devices to move beyond high‐end research settings. It is recommended that in future studies the model number of the device, sensors, technical data and the software version utilised with the device is clearly documented. Although studies which correlate devices appear to be popular they should also include data on measurement error. As TEWL measurement devices result in continuous scores the recommended statistics according to Mokkink et al.^13^ to measure reliability is the use of ICCs but also to note the model utilised. For measurement error CV, SEM, SDC or LoA should be utilised and also have the model specified and match the data.

Consideration of assessors administering the measurement and/or assigning the scores should be noted in future studies, or in the very least, this risk of bias should be noted and strategies employed to reduce the risk However, as TEWL devices tend to be considered an objective measurement tool the risk of bias by having the same investigator complete the measures, repeated measures and record the data is minimal. As noted by many of the authors in the included studies and by other review articles, although devices correlate, they do not produce the same values. Studies which include different devices cannot be compared against each other.

An additional consideration for the user of TEWL measurement devices is the variability external to the device. In a review by Peer et al.^54^ they identified variables such as age, anatomic site and temperature should be controlled for in TEWL studies. Other confounding variables discussed was technician training, room temperature, season, sleep, certain foods, eccrine sweating and body mass index.^54^ Controlling for these experimental, environmental and individual variables needs to also be taken into consideration when examining and conducting studies measuring TEWL.

A limitation of this systematic review was perhaps the choice of the risk of bias tool for critical appraisal. This tool includes only six questions on study quality and did not suit study design as many did not have blinding of raters and those assigning the scores. This component of the study may not be as relevant where an objective device is being examined where the assignment of scores is not dependent on the rater. If selection of location to place the device to take a TEWL measurement is clearly defined then blinding of the rater would also not be as great importance. This risk of bias tool has only just been released and has been adapted from analysis of PROMs. It will almost certainly evolve.

In conclusion, we have been able to systematically obtain studies relevant to the inclusion criteria, analyse the measurement properties and synthesise the information. The use of reliability and measurement error statistics on average are doubtful. Many devices are able to be correlated to each other but no ‘gold standard’ has been elucidated. Accuracy of TEWL measurement devices increases in in vitro settings as expected. Future research should consider risk of bias factors when designing studies however the risk of bias tool utilised in this systematic review has only recently been developed and will likely be developed further.

## CONFLICTS OF INTEREST

The authors have no known conflicts of interest to declare.

## APPENDIX 1 Search strategy

Search algorithm examples:

### PubMed

(((“Skin”[MeSH Terms] OR “Skin”[Text Word]) AND (((“transepidermal water loss”[Text Word] OR “TEWL”[Text Word]) OR “water loss, insensible”[MeSH Terms]) OR “water loss insensible”[Text Word])) AND ((“instrumentation”[MeSH Subheading] OR “equipment and supplies”[MeSH Terms]) OR “Device”[Text Word] OR “evaporimeter”[Text Word])) AND “English”[Language]

### CINAHL

(MW Skin OR TI Skin OR AB Skin OR TI Epidermis OR AB Epidermis OR AB Dermis OR TI Dermis) AND (MH Water loss, insensible OR TI Water loss, insensible OR AB Water loss, insensible OR TI transepidermal water loss OR AB transepidermal water loss OR TI TEWL OR AB TEWL OR TI Insensible water loss OR AB Insensible water loss OR TI IWL OR AB IWL OR TI TWL OR AB TWL) AND (MH Research instruments OR TI Research instrument* OR AB Research instrument* OR MH equipment and supplies OR TI equipment and suppl* OR AB equipment and suppl* OR TI Device OR AB Device OR TI Evaporimeter OR AB Evaporimeter)

Narrowed by language: English.

### Embase

(skin:de,ti,ab OR epidermis:ti,ab OR dermis:ti,ab) AND (‘skin water loss’:de,ti,ab OR ‘transepidermal water loss’:ti,ab OR tewl:ti,ab OR ‘insensible water loss’:ti,ab OR twl:ti,ab OR iwl:ti,ab) AND (instrumentation:ti,ab OR equipment:ti,ab OR devices:de,ti,ab OR evaporimeter:ti,ab) AND [english]/lim

## APPENDIX 2 Risk of bias assessment tool^13^

Standards for design requirements of studies on reliability or measurement error

**Table:**

Design requirements	Very good	Adequate	Doubtful	Inadequate	NA
1. Were patients stable in the time between the administration of the repeated measurements on the construct to be measured?	Yes (evidence provided)	Reasons to assume standard was met	Unclear if standard was met	No	NA
2. Was the time interval between the measurements appropriate?	Yes		Doubtful if standard was met OR time interval not stated	No	NA
3. Were the measurement conditions similar for the measurements—except for the condition being evaluated as a source of variation?	Yes (evidence provided)	Reasons to assume standard was met, OR change was unavoidable	Unclear if standard was met	No	NA
4. Did the professional(s) administer the measurement without knowledge of scores or values of other repeated measurement(s) in the same patients?	Yes (evidence provided)	Reasons to assume standard was met	Unclear if standard was met	No	NA
5. Did the professional(s) assign the scores or determined the values without knowledge of the scores or values of other repeated measurement(s) in the same patients?	Yes (evidence provided)	Reasons to assume standard was met	Unclear if standard was met	No	
6. Were there any other important flaws in the design or statistical methods of the study?	No		Minor methodological flaws	Yes	

Standards for preferred statistical methods for reliability

**Table:**

Statistical methods	Very good	Adequate	Doubtful	Inadequate
7a. For continuous scores: was an ICC calculated?	ICC calculated; the model or formula was described, and matches the study design and the data	ICC calculated but model or formula was not described or does not optimally match the study design OR Pearson or Spearman correlation coefficient calculated WITH evidence provided that no systematic difference between measurements has occurred	Pearson or Spearman correlation coefficient calculated WITHOUT evidence provided that no systematic difference between measurements has occurred OR WITH evidence provided that systematic difference between measurements has occurred	
8a For ordinal scores: was a (weighted) Kappa calculated?	Kappa calculated; the weighting scheme was described, and matches the study design and the data	Kappa calculated, but weighting scheme not described or does not optimally match the study design		
9a. For dichotomous/nominal scores: was Kappa calculated for each category against the other categories combined?	Kappa calculated for each category against the other categories combined			

*Note*: Grey shading—these questions were not utilised in this systematic review as all transepidermal water loss (TEWL) devices produce continuous scores.

Abbreviation: ICC, intra‐class correlation coefficient.

Standards for preferred statistical methods for measurement error (agreement)

**Table:**

Statistical methods	Very good	Adequate	Doubtful	Inadequate
7b. For continuous scores: was the SEM, SDC, LoA or CV calculated?	SEM, SDC, LoA or CV calculated; the model or formula for the SEM/SDC is described; it matches the study design and the data	SEM, SDC, LoA or CV calculated, but the model or formula is not described or does not optimally match the study design and evidence provided that no systematic difference has occurred	SEM consistency SDC consistency or LoA or CV calculated, without knowledge about systematic difference or with evidence provided that systematic difference has occurred	SEM calculated based on Cronbach's alpha OR Using SD from another population
8b. For dichotomous/nominal/ordinal scores: was the percentage specific (e.g. positive and negative) agreement calculated?	% specific agreement calculated	% agreement calculated		

*Note*: Grey shading—these questions were not utilised in this systematic review as all transepidermal water loss (TEWL) devices produce continuous scores.

Abbreviations: CV, coefficient of variation; LoA, limits of agreement; SD, standard deviation; SDC, smallest detectable change; SEM, standard error of measurement.

## APPENDIX 3 Excluded studies with reasons

### 3.1

**Table:**

Author	Title	Reasons
Bak et al.^58^	Portable device, based on a microcontroller, for measurement of TEWL factor	Describes methods of experiments to test devices that measure TEWL. Is not a comparison of devices.
Baker and Kligman^59^	Measurements of TEWL by electrical hygrometry: instrumentation and responses to physical and chemical insults	Measurements taken under different conditions but no measurements for purposes of measuring reliability or comparison with another device.
Cohen et al.^60^	Comparison of closed and open chamber evaporimetry	Does not report on correlation of data obtained from the two devices or intra‐ or inter‐reliability. Measures effect of change of measuring angle.
Imhof et al.^61^	Closed chamber TEWL measurement: microclimate, calibration and performance	Review article.
Petro and Komor^62^	Correction to absolute values of evaporation rates measured by the Servo‐Med Evaporimeter	Describes adjustments that need to be made to this specific device's TEWL measurements that were previously inaccurate at higher rates of TEWL. Does not include reliability data or comparison to other devices.
Valentin et al.^63^	A novel TEWL sensor	No reliability or measurement error data contained within the article.
Weigmann et al.^64^	Comparison of TEWL and spectroscopic absorbance to quantify changes of the stratum corneum after tape stripping	Does not include reliability data or comparison to other devices for TEWL measurement. TEWL was used as a measure of SC thickness.

Abbreviation: TEWL, transepidermal water loss.

## APPENDIX 4 Included studies

### 4.1

**Table:**

Author	Title	No. of subjects	Study population/method	In vivo or in vitro	Device/s	Results: measurement property—measurement error/agreement	Results: measurement property—reliability
Anthonissen et al. ^51^	Measurement of elasticity and transepidermal water loss rate of burn scars with the DermaLab	32	Thirty‐two active burns scars (grafted and spontaneously healed) and normal skin. Two consecutive measurements for TEWL were taken by a first assessor and one measurement by a second observer. A period of 4 min between all measurements was taken.	In vivo	DermaLab (Cortex Technologies, Hadsund, Denmark)	SEM ≤1.74 for intra‐observer reliability and SEM ≤2.76 for inter‐observer reliability.	Intra‐ and inter‐observer reliability. ICC ≥0.86 for intra‐observer reliability and ICC ≥0.78 for inter‐observer reliability.
Barel and Clarys^55^	Study of the stratum corneum barrier function by transepidermal water loss measurements: comparison between two commercial instruments—Evaporimeter and Tewameter	16	Volunteers aged 18–30 years. Various anatomical sites. Conditions: normal, after occlusion or stripping.	In vivo	Evaporimeter 2100 (Servomed AB, Stockholm, Sweden), Tewameter TM210 (Courage and Khazaka, Cologne, Germany)	The range of the CV for the Tewameter varies from 6% to 13% and the Evaporimeter varies from 3% to 6% at different anatomical sites.	Influence of external room temperature, *r* = 0.88 for Tewameter and *r* = 0.89 for Evaporimeter, correlates with increase in temperature. There is a significant effect of the pressure of the probe on the skin. Significant air movements have an effect on TEWL measurements but breathing or moving doors has almost no influence. Correlation between TEWL measurements at different skin sites under various conditions, *n* = 16, *r* = 0.97.
Blichmann and Serup^37^	Reproducibility and variability of transepidermal water loss measurement	10	Ten healthy volunteers. Measurements done on palm of the hand and flexor side of forearm. Successive measurements on the same individual over 3 min.	In vivo	Evaporimeter EP1 (Servomed AB)	Intra‐individual reproducibility on palm of hand: CV = 9.4%. Intra‐individual reproducibility on the forearm = 9.1%. Variation of TEWL among different individuals to measurements on the palm and forearm show a CV ranging from 31% to 57%. The variation by time of the group of individuals (over 24 h) was relatively minor.	Intra‐individual reproducibility reported as mean difference as % of initial TEWL measurements on the palm of the hand varied between 6.4% and 14.5% and for the forearm varied between 11.3% and 34.1%.
Cointereau‐Chardon et al.^49^	Self‐recording the skin hydration and transepidermal water loss parameters: a pilot study	20	Twenty healthy women recruited through an employment agency. Selected as they had moderately dry skin on cheeks and forearms. Aged 45–60 years. Two successive measurements were self‐recorded and in the lab on two close regions on cheeks and forearm (four sites). Then applied glycerol product. Measured every hour until 6 h and again in the morning. Then after washing and 15 min later.	In vivo	GPSkin Pro (Gpower, Seoul, South Korea)	TEWL arm: mean = 2.96, SD = 4.49. TEWL face: mean = 3.85, SD = 7.06.	
De Paepe et al.^9^	Validation of the Vapometer, a closed unventilated chamber system to assess transepidermal water loss versus the open chamber Tewameter	16	Sixteen healthy females, no skin disease. Evaluation of skin on forearms. Six visits over a 4 weeks period. Measurements: baseline values, reproducibility assessed by conducting 10 consecutive measurements per volunteer, effect of an application of a cosmetic cream and recovery after SLS. Corresponding test zone on other forearm treated with water.	In vivo	Tewameter TM210 (Courage and Khazaka), Vapometer SWL‐2 (Delphin Technologies, Kuopio, Finland)	The variability of the baseline readings for each site was similar for both devices. Values significantly higher (*p* < 0.001) for the Tewameter than for the Vapometer. Smaller differences in TEWL could be detected with the Tewameter at tape stripping skin. Based on the standard errors of the differences and the mean square error from the analysis of variance, the Tewameter provided more precise results when measuring the effect of a moisturiser. TEWL values obtained with the Vapometer, 10 consecutive measurements for each volunteer. A shift in an upward direction was observed going from 5 ± 3 g/h/m^2^ (first replicate) to 7 ± 3 g/h/m^2^ (10th replicate). The variability of each replicate, however, remained the same (Levene's test for equality of variances, *p* = 0.778). When the higher sets of readings were compared with the first set, a significant difference was found for the fifth and the seventh reading onwards, meaning that reproducible results were only obtained for the first four sets of readings.	Spearman's correlation coefficients were determined, the results retrieved with both devices were correlated (*r* varying from 0.503 to 0.966).
Elkeeb et al.^30^	Correlation of transepidermal water loss with skin barrier properties in vitro: comparison of three evaporimeters		Dermatomed human cadaver skin without obvious signs of skin disease. TEWL measurements were carried out by placing the collared probe over the specially designed top of the donor compartment 1 cm away from the surface of the skin, and was fitted using a surrounding rubber ring. Measurements were conducted at 0 times (before 3 h water dosing) and 1, 2, 4 and 24 h after dosing.	In vitro	Tewameter TM210 (Courage and Khazaka), AquaFlux AF200 (Biox Systems Ltd, London, UK)		The patterns of TEWL profiles from the three instruments were similar. However, the measurement capacity of the AquaFlux is significantly higher than those of Tewameter or Vapometer (value not given, *p* < 0.001). Baseline TEWL measured with AquaFlux at zero time is correlated with the flux rate of tritiated water (*p* = 0.04, *r* ^2^ = 0.34). Baseline TEWL measured with Tewameter, similar to that of AquaFlux, correlates with the flux of tritiated water (*p* = 0.00, *r* ^2^ = 0.50). Vapometer, however, shows no statistically significant correlation with the flux rate of tritiated water (*p* = 0.07, *r* ^2^ = 0.31).
Farahmand et al.^33^	Measuring transepidermal water loss : a comparative in vivo study of condenser‐chamber, unventilated chamber and open ‐chamber systems	6	Performed on human forearm skin (*n* = 6) without skin disorders, in the normal condition (baseline), and after (1) 10 tape strippings on both arms, (2) moisturiser cream (Eucerin Calming Cream) and petrolatum application for 1 h and (3) 1% SLS aqueous solution and distilled water (as control) application for 20 min. All measures performed three times.	In vivo	Tewameter TM210 (Courage and Khazaka), Vapometer SWL‐2 (Delphin Technologies), AquaFlux AF200 (Biox Systems Ltd)	Tewameter CV ranged from 17.3% to 60%. Vapometer CV 11.5%–49.6% AquaFlux CV 4.8%–31.1%	TEWL values measured by three instruments are correlated significantly (*p* < 0.001). Pearson's correlation coefficient is 0.58 for AquaFlux and Vapometer, 0.70 for Vapometer and Tewameter, and 0.88 for Tewameter and AquaFlux.
Fell et al.^52^	The Scarbase Duo: intra‐ and inter‐rater reliability and validity of a compact dual scar assessment tool	20	Twenty patients (20 scars) recruited from burns outpatient clinic with active scars. Scars on the upper and lower limbs. Average scar age 5.65 m. Contralateral area of skin used for comparison or neighbouring skin. Two measurements taken by the first rater with Scarbase Duo then measured the same area with the DermaLab. The second rater conducted a single measure with each device.	In vivo	DermaLab (Cortex Technologies), Tewameter (as part of Scarbase Duo: Courage and Khazaka)	Moderate SEM values (0.74–1.17) for Tewameter. The Bland–Altman plots for agreement between the two tools on scars show the bias of the mean is high, suggesting that a systematic error could be detected. The DermaLab systematically measures approximately 2.5 g/m^2^ higher than the Tewameter. The LoA are far apart, suggesting that the high correlation between the two measurement methods is not supported by high agreement.	Tewameter: intra‐rater reliability (scar), ICC = 0.95, SEM = 1.17, (healthy skin), ICC = 0.87, SEM = 0.74; inter‐rater reliability (scar), ICC = 0.96, SEM = 1.12, (healthy skin), ICC = 0.90, SEM = 0.75. Concurrent validity of TEWL measurements between the Tewameter and the DermaLab—scar: ICC = 0.81, *r* = 0.93, good to excellent correlation. Healthy skin: ICC = 0.52, *r* = 0.72, moderate correlation. The DermaLab systematically measures approximately 2.5 g/m^2^ higher than the Tewameter.
Fluhr et al.^7^	transepidermal water loss reflects permeability barrier status: validation in human and rodent in vivo and ex vivo models		Ex vivo mouse model: skin removed from mice. In vivo mouse model: lowering of TEWL with petrolatum, increase TEWL by tape stripping 2, 3, 6 and 8×. In vivo human skin: volar forearms, tape stripped skin 10×.	Ex vivo, in vivo	DermaLab (Cortex Technologies), Evaporimeter EP1 (Servomed AB), Tewameter TM210 (Courage and Khazaka), Tewameter TM300 (Courage and Khazaka), Vapometer (Delphin Technologies), H4300 (NIKKISO‐YSI Co, Ltd, Tokyo, Japan), MEECO (MEECO, Warrington, PA, USA)	The CV was highest for the DermaLab (18.0), followed by MEECO (16.6), Vapometer (12.8) (in vivo). The H4300 (12.4), TM210 (12.0), EP1 (13.5) and TM300 (1.9): in vivo study on human ventral forearm.	Correlation with gravimetric values: for EP1 (*r* = 0.8076) > TM210 (*r* = 0.7666) > VapoMeter (*r* = 0.7630) > TM300 (*r* = 0.7557) > H4300 (*r* = 0.7082) > MEECO (*r* = 0.6825). Significant correlation was found between all devices of at least *r* > 0.83 however the TM210 correlated less well than the other devices (*r* up to 0.9879). All instruments significantly correlated with each other from the in vivo study on the ventral forearms, however, the MEECO system correlated less well with the H4300 and the TM210.
Gardien et al.^21^	transepidermal water loss measured with the Tewameter TM300 in burn scars.	53	Fifty‐five (final 53, two excluded due to instrument reading error) patients with burn scars 3, 6 and 12 m after burn. Morphological characteristics measured with POSAS and colour measured with DSM II ColourMeter. Three study areas: scar, adjacent normal skin and contralateral skin. Two observers. Two measures by first observer and one measure by second observer. 94% of observer 1 measurements were one person. Single measurements were performed by two different observers 42% and 58%.	In vivo	Tewameter TM300 (Courage and Khazaka)	Intra‐observer SEM: 2.38–2.68 g/m^2^/h. Inter‐observer SEM: 1.76–3.97 g/m^2^/h. Bland–Altman plots showed relatively wide LoA values for scar and healthy skin.	Intra‐observer reliability ICC: 0.89–0.92 (excellent agreement). Inter‐observer reliability ICC: 0.85–0.94 (excellent agreement).
Grinich et al. ^45^	Validation of a novel smartphone application‐enabled, patient‐operated skin barrier device	50	Fifty participants with healthy skin, volar forearms. Participants collected their own measurements with GPSkin with minimal instruction. AquaFlux measurements were taken by the investigator. Because of the low test‐‐retest reliability in the first trial, methods were modified to include increased participant education on device use prior to the start of trial 2.	In vivo	GPSkin Pro (Gpower), AquaFlux AF200 (Biox Systems Ltd)		In trial 1, GPSkin demonstrated ‘poor’ test‐‐retest reliability (ICC = 0.18, 95% CI: −0.08 to 0.42) for TEWL, whereas the AquaFlux showed ‘moderate’ reliability (ICC = 0.58, 95% CI: 0.36‐‐0.73) for TEWL. In trial 2, test‐‐retest reliability of GPSkin TEWL measurements improved to ‘good’ for participant (ICC = 0.89, 95% CI: 0.82‐‐0.94), investigator (ICC = 0.88, 95% CI: 0.79‐‐0.93) and participant‐‐investigator (ICC = 0.88, 95% CI: 0.79‐‐0.93) comparisons. The AquaFlux also demonstrated ‘good’ reliability for TEWL (ICC = 0.86, 95% CI: 0.76‐‐0.92). The devices were moderately correlated by Spearman's for TEWL (*r* ^2^ = 0.48, *p* = 0.0004) when AquaFlux versus participant taken measures with GPSkin. Participant GPSkin after education TEWL measurements and the AquaFlux were moderately correlated (*r*s = 0.40, *p* = 0.0045) while investigator GPSkin TEWL measurements and the AquaFlux were weakly correlated (*r*s = 0.34, *p* = 0.0147).
Grinich et al.^46^	Validation of a novel patient‐operated device for measuring skin barrier function in atopic dermatitis	50	Fifty 18+ years of AD patients with varying disease severity performed self‐measurements at volar forearm with GPSkin, while investigators collected data, two measurements with each, with all three devices, on both non‐lesional and lesional skin.	In vivo	GPSkin Pro (Gpower), AquaFlux AF200 (Biox Systems Ltd)	GPSkin demonstrated poor agreement with standard devices in Bland–Altman plots. GPSkin consistently measured lower mean values for TEWL compared to the AquaFlux as indicated by the positive bias line (mean difference between measurements from GPSkin and the standard device). The discrepancy in GPSkin and standard devices was most significant at higher TEWL values.	GPSkin versus AquaFlux demonstrated strong correlation for patient measured: *r* ^2^ (non‐lesional) = 0.72, *r* ^2^ (lesional) = 0.68 and clinician measured *r* ^2^ (non‐lesional) = 0.80, *r* ^2^ (lesional) = 0.66. Test–retest reliability ICC values for GPSkin ranged from 0.67 to 0.90 (CIs provided in the study). The AquaFlux ICC value was 0.90 (0.84–0.94) for non‐lesional skin and 0.76 (0.61–0.86) for lesional skin.
Grove et al.^38^	Comparative metrology of the Evaporimeter and the DermaLab transepidermal water loss probe	11	In vitro: foam fully loaded with standard amount of water placed under probes simultaneously under different polymeric films with different WVTR. A series of measurements in which the evaporative water loss was sequentially determined on the low, medium and high WVTR films 10 times in succession was performed using a fixed duration program that calculated the mean water loss rate. In vivo: 11 panellists had volar forearms exposed to 5% aqueous solutions of eight test products under occlusion for 24 h. TEWL rates measured in duplicate by Evaporimeter and DermaLab.	In vitro and in vivo	DermaLab (Cortex Technologies), Evaporimeter EP1 (Servomed AB)	The CV was higher for the Servo Med evaporimeter (6.2%–46.8%) than the DermaLab (4.3%–20.7%), which means the reproducibility was better with the DermaLab (in vitro).	There was excellent agreement between the Servo Med evaporimeter and the DermaLab TEWL probe (*R* ^2^ = 0.9589). The DermaLab values tended to be less than the corresponding value obtained with the Servo Med evaporimeter. There was excellent agreement between the Servo Med evaporimeter and the DermaLab in vivo (*R* ^2^ = 0.8665).
Hon et al.^22^	Are skin equipment for assessing childhood eczema any good?	80	Subjects with AD, aged 1–18 years, measurements at 2 cm below elbow, volar forearm. Eighty sets of clinical scores.	In vivo	Tewameter TM300 (Courage and Khazaka), Vapometer (Delphin Technologies)		There are significant correlations between measurement by the two devices of TEWL (*r* = 0.357, *p* < 0.05). However, it seems that Vapometer performs better than TM300 in general with regards to comparison to other clinical measures.
Hua et al.^23^	Comparison of two series of non‐invasive instruments used for the skin physiological properties measurements: the DermaLab from Cortex Technology versus the series of detectors from Courage and Khazaka	30	Thirty healthy volunteers, areas on the face measured. Randomly assigned to Group A or B where instruments were swapped with regards to which side they measured. Also measured on mid forearm. Three measurements on each side.	In vivo	DermaLab (Cortex Technologies), Tewameter TM300 (Courage and Khazaka)	DermaLab TEWL probe CV% minimum–maximum 0.00–20.75, mean = 6.35, SD = 3.92. TM300 CV% 0.86–17.70, mean = 6.08, SD = 3.57.	DermaLab versus TM300 *r* = 0.940, *p* < 0.001
Imhof et al.^31^	Rapid measurement of transepidermal water loss with a condenser‐chamber instrument	1	A condenser‐chamber device (AquaFlux) was compared to a traditional open chamber device (Vapometer). In vivo studies used 12 measurements from seven sites (wrist to forearm) with both devices. In vitro study used 200 repeat measurements from each device.	In vivo and in vitro	Vapometer (Delphin Technologies), AquaFlux AF200 (Biox Systems Ltd)	For AquaFlux TEWL measurements in vivo SD varied between 0.81 g/m^2^/h, CV = 6.4% at wrist and SD 0.16 g/m/h, CV = 2.0% in middle of forearm. Compared to SD 0.09 g/m/h and CV 1.2% in vitro. For the Vapometer results from 200 measurements using an in vitro source had an average of 9.02 g/m^2^/h and a CV of 10.3%. The Vapometer in vivo source CV varied from 8.2% to 12.3%.	There is broad agreement between the VapoMeter and AquaFlux measurements, characterised by a Pearson correlation coefficient of *R* = 0.82 (in vivo).
Kikuchi et al.^24^	Comparison of the measuring efficacy of transepidermal water loss of a reasonably priced, portable closed chamber system device H4500 with that of rather expensive, conventional devices such as Tewameter and Vapometer	15	Ten healthy volunteers. Measurements conducted by the same investigator, repeated 10 times on volar forearm (Study 1). Study 2: 15 healthy volunteers, forearms, taped stripped on the right and 0.5% SLS applied for 24 h under occlusion on the left.	In vivo	Tewameter TM300 (Courage and Khazaka), Vapometer SWL‐2 (Delphin Technologies), H4500 (Nikkiso‐Therm Co Ltd, Musashino, Japan)		Intra‐rater reliability: ICC with 95% CI of H4500, Vapometer and Tewameter were 0.927 (0.835–0.978), 0.856 (0.697–0.955) and 0.93 (0.842–0.979), respectively. All measured sites: H4500 versus Tewameter *r* = 0.945, H4500 versus Vapometer *r* = 0.910, Vapometer versus Tewameter *r* = 0.939 (all *p* < 0.001). Healthy skin: H4500 versus Tewameter *r* = 0.756, H4500 versus Vapometer *r* = 0.431, Vapometer versus Tewameter *r* = 0.492. Immediately after tape stripping: H4500 versus Tewameter *r* = 0.718, H4500 versus Vapometer *r* = 0.900, Vapometer versus Tewameter *r* = 0.850. 24 h after tape stripping: H4500 versus Tewameter *r* = 0.811, H4500 versus Vapometer *r* = 0.843, Vapometer versus Tewameter *r* = 0.861. After 24 h of SLS: H4500 versus Tewameter *r* = 0.768, H4500 versus Vapometer *r* = 0.849, Vapometer versus Tewameter *r* = 0.533.
Lau‐Gillard et al.^36^	Evaluation of a hand held evaporimeter (VapoMeter) for the measurement of transepidermal water loss in healthy dogs	23	One human volunteer and 22 dogs. All healthy without skin conditions. Measurements carried out by a single operator in non‐climate controlled room but stable conditions. Human participant uncovered skin immediately before readings. TEWL measured 10× at four different body sites on human. Upright and upside‐down. Repeated measurements 4 days later. In dogs: 10 readings on unclipped skin and 2 min after clipping. Ten consecutive readings obtained at 12 body sites on 5 different days (non‐consecutive) in both the short‐coated dog and the long‐coated dog. To compare TEWL in a larger group of dogs, 10 consecutive readings obtained from the right and left lateral thorax in the additional 20 dogs.	In vivo	Vapometer SWL‐5 (Delphin Technologies)	For the short‐coated dog, CVs for readings obtained at various sites on various days ranged from 7.3% to 76.9% with a mean of 33.4% (95% CIs ranged from 29.5% to 37.2%). Long‐coated dog, CVs ranged from 4.7% to 43% with a mean of 20.3% (95% CIs ranged from 18% to 22.7%). CVs were significantly lower in both axillae compared to the top of head and between shoulders, and on the right leg compared to between shoulders (Tukey's multiple comparison, *p* < 0.05).	
Logger et al.^47^	Value of GPSkin for the measurement of skin barrier impairment and for monitoring of rosacea treatment in daily practice	43	Pilot 1: 27 healthy participants. GPSkin compared with AquaFlux at the forearm before and after tape stripping and at both cheeks without intervention. Pilot 2: 16 rosacea patients, GPSkin measurements at the forearm and both cheeks before and during anti‐inflammatory treatment, values compared to pilot 1 values.	In vivo	AquaFlux AF200 (Biox Systems Ltd)		Pilot 1: GPSkin versus AquaFlux: linear regression *R* = 0.8718, *p* < 0.0001, Spearman correlation coefficient *R* = 0.9256, *p* < 0.0001
Miteva et al.^25^	Approaches for optimising the calibration standard of Tewameter TM300		Under laboratory conditions consecutive measurements taken with five different probes on three different membrane surfaces.	In vitro	Tewameter TM300 (Courage and Khazaka)	The results obtained by measuring with five different probes on three different types of models (i.e. a heated petri dish, an unheated petri dish and a calibration bottle) revealed some variation. SDs varied from 1% to 6% for the heated petri dish and 2%–9% for the unheated petri dish. The SD for the measurements completed on the calibration bottle reached a maximal of 0.4%.	
Murphrey et al.^48^	Can a hand held device accurately measure barrier function in ichthyoses?	30	Thirty ichthyosis subjects and 25 age and sex matched controls. Three serial TEWL readings taken at non‐overlapping locations on the volar arm. Taken on 2 successive days.	In vivo	GPSkin Barrier Light (Gpower), AquaFlux AF200 (Biox Systems Ltd)	Bland–Altman analysis was performed to assess agreement between AquaFlux and GPSkin, which was worse at higher TEWL values for GPSkin.	In the combined cohort, both devices showed excellent test–retest reliability, with ICC = 0.984 (95% CI, 0.973–0.991) for AquaFlux and ICC = 0.974 (95% CI, 0.959–0.984) for GPSkin. When the population was analysed after sub‐dividing into ichthyosis and controls, the ICCs remained excellent for subjects with ichthyosis (AquaFlux ICC = 0.976 (95% CI, 0.954–0.988), GPSkin ICC = 0.974 (95% CI, 0.953–0.987)), and was good for controls (AquaFlux ICC = 0.816 (95% CI, 0.646–0.912), GPSkin ICC = 0.868 (95% CI, 0.745–0.937)). Readings from the two instruments were strongly positively correlated for the entire cohort (*r*s = 0.743, *p* < 0.001) and moderately well correlated when analysed separately for ichthyosis (*r*s = 0.518, *p* = 0.003) and controls (*r*s = 0.536, *p* = 0.006). When the entire population was divided at the median of GPSkin measurements, the correlation between GPSkin and AquaFlux was better at the higher range (above the median, 9.7 g/m^2^/h; *r*s = 0.675, *p* < 0.001) than below the median (*r*s = 0.499, *p* = 0.008), although correlation was still moderate to strong with both devices.
Norlén et al.^56^	A new computer‐based evaporimeter systemic for rapid and precise measurements of water diffusion through stratum corneum in vitro	36	A new computer‐based evaporimeter was used to measure water uptake, water diffusion rate under controlled conditions in vitro stratum corneum samples from 36 patients who had breast reduction. Measurements were taken over 10 000 s to achieve equilibrium. The system was used on a free water surface, with 20 runs to gain reference data. Water diffusion data were calculated 10 times (once every 3 days) in vitro on one SC piece. This was compared to 10 measurements of one patient from mid forearm.	In vitro	Author fabricated evaporimeter, Evaporimeter 2100 (Servomed AB)	The precision of the water diffusion rate measurements through mounted SC samples was 11% (TEWL being 1.9 ± 0.1 g/m^2^/h (95% CI of mean; *n* = 10)), the corresponding value for the TEWL measurements in vivo on human left forearm was 36% (TEWL being 8.7 ± 1.9 g/m^2^/h (95% CI of mean; *n* = 10).	
Nuutinen et al.^32^	A closed unventilated chamber for the measurement of transepidermal water loss.	10	Water‐filled petri dish with a semi‐permeable membrane heated to 80°C then allowed to cool. Measurements taken during cooling process. Dish weighed with a scale simultaneously. Ten health volunteers had TEWL measurements repeated 10 times on volar forearm and palm of the hand.	In vitro and in vivo	Vapometer (Delphin Technologies)	For the Vapometer only: the CV was 8.0% for the forearm, 10.1% for the palm of the hand and 4.0% for the petri dish.	Evaporation rate from a petri dish was correlated with the Vapometer and DermaLab measurements. Evaporation rate of Vapometer was *r* = 0.99, *p* < 0.001 until the evaporation was 200 g/m^2^/h from the petri dish. For the DermaLab up to 120 g/m^2^/h, *r* = 0.99, *p* < 0.001, after this it underestimates evaporation rates
Park and Tamura^39^	Measurement of regional evaporation rate from skin surface by evaporimeter	10	After calibration of two sensors measurements were taken at the thigh with and without a mounted wetted filter paper. Measurements confirmed by weighing the wetted filter paper every 30 min and comparing total weight loss of the person. Assessments also completed at different temperatures. The probe was applied on each skin/wetted paper surface every 5 s for 10 min. Subjects were 10 female students aged 22—34 years.	In vitro and in vivo	Evaporimeter EP1 (Servomed AB)		Correlation between evaporation rate measured and weight loss of filter paper, *r* = 0.98. Correlation between body weight loss and cutaneous evaporation rate, *r* = 0.91. Regression formula for body weight loss (*y*) compared with evaporation rate (*x*) is *y* = 2.06 + 2.53*x*.
Pinnagoda et al.^40^	Comparability and reproducibility of the results of water loss measurements: a study of four evaporimeters	1	In vitro: petri dish filled with water and covered with Opsite dressing to form a semi‐permeable membrane. Plastic cover pulled away so probe can begin measuring from 3 mm away for 6 min, 5× per probe. In vivo: TEWL measured on volar aspect of the right forearm of one subject at one location. Recorded continuously for 4 min, 3× measurements per probe.	In vitro and in vivo	Evaporimeter EP1 (Servomed AB)	In vitro: after stabilisation the SDs of the mean for each probe decreased from 0.8 to 0.2 g/m^2^ h indicating reproducibility of the successive measurements is high. In vivo: until stabilisation was reached the SDs of the mean for each probe decreased from about 0.4 to 0.2 g/m^2^ h. After stabilisation, they remained below 0.2 g/m^2^ h.	
Rogiers^41^	Transepidermal water loss measurements in patch test assessment: the need for standardisation	21	The Tewameter TM200 was compared to the Evaporimeter EP1 in 21 females (22–29). TEWL measurements were measured and compared under various conditions (different probe temperatures, different skin hydration, environmental temperature and patch test with blank and SLS patches).	In vivo	Evaporimeter EP1 (Servomed AB), Tewameter TM200 (Courage and Khazaka)	For blank patch comparison Tewameter had TEWL of 8.1 ± 1.2 left arm and 8.0 ± 1.1 right arm as opposed to EP1 values of 4.6 ± 1 left arm and 4.9 ± 1.1 right arm. No significance was found in difference between sites, but EP1 had significantly lower values (*p *< 0.001) in both sites. For blank versus SLS patches the values for TM200 were 30.3 ± 11.6 (CV = 38%) versus 8.1 ± 1.4 (CV = 17%), respectively. The respective values for EP1 were 18.9 ± 8.5% (CV = 45%) versus 4.6 ± 1.2 (CV = 26%). The difference in values between blank and SLS was significant between the two groups. The intergroup CV was significantly higher for the EP1. Inter‐group variations (10.8 ±3.5) and intra‐group (20.1 ± 2.7) variability for measurements at different times of the day and on the same day across different weeks were not significant.	
Rosado et al.^26^	Comparative assessment of the performance of two generations of Tewameter: TM210 and TM300	15	Fifteen healthy volunteers. Measurements taken on the volar forearm. Comparative measurements with the two devices were carried out simultaneously, on opposite sites in each volar forearm. Recorded components: stabilisation time, TEWL, VC% after three TEWL measurements performed every 5 min, VC% in extreme conditions (three measurements every 5 min after the skin was covered with an occlusive patch for 1 h).	In vivo	Tewameter TM210 (Courage and Khazaka), Tewameter TM300 (Courage and Khazaka)	Under normal conditions the VC% was not statistically significantly different (*p* = 0.27) but the TM210 performed slightly better with an average VC of 6.75. Under extreme conditions the TM300 performed better but was not statistically significantly different (*p* = 0.310). For t1/2evap (evaporation half‐life) the TM210 provided higher TEWL results and slower decay and was statistically different from the TM300 (*p* = 0.003). The measurement of dynamic water mass also differed between the two models (*p* = 0.003).	
Scott et al.^42^	A comparison of techniques for the measurement of transepidermal water loss	72	Normal and tape stripped rat skin measured first with Evaporimeter then with a purpose built ventilated chamber apparatus. Measurements taken on 72 animals.	In vivo	Evaporimeter EP1 (Servomed AB)	Untreated skin: mean TEWL values was 0.30, SEM ±0.02 and for the unventilated chamber was 0.32, SEM ±0.02. Tape stripped skin (*n* = 9): evaporimeter mean TEWL 7.41, SEM ±0.16, ventilated chamber 19.04 ±SEM 1.60.	
Shah et al. ^34^	Comparative evaporimetry in man	9	Two Tewameters and one Vapometer used on nine healthy volunteers with normal skin. Three sets of TEWL measurements performed simultaneously with each instrument. Seven sites chosen: three forehead, three volar forearm, and middle of the scalp (Vapometer only).	In vivo	Tewameter TM210 (Courage and Khazaka), Vapometer SWL‐3 (Delphin Technologies)	In all cases the SDs associated with each instrument were small and the CV for all sites clearly indicated that the reproducibility of successive measurements with any individual instrument was high. CV for TM210 ranged from 9.2% to 25%. CV for the Vapometer ranged from 14.9% to 21.2%.	For TEWL values of all forearm locations, there were no statistically significant differences between the mean values measured by all three instruments (*p* = 0.68–0.90). For TEWL values of forehead locations, there was significant difference between the mean values of both open chamber device and closed chamber device (*p* = 0.0049–0.04).
Sim et al. ^50^	Portable skin analysers with simultaneous measurements of transepidermal water loss, skin conductance and skin hardness		Device (THC) calibrated using a wet‐cup method: water‐filled petri dish is covered with a semi‐permeable membrane, controlled for temperature of the water. Evaluated in terms of sensitivity and linearity from the measured calibration curve. Compared against the GPSkin.	In vitro	GPSkin Pro (Gpower), THC	THC sensitivity of 0.0068 (%/s)/(g/m^2^/h) with the high linearity of 99.63%. Coefficient of determination shows 0.8613, indicating a good agreement of a linear relation between two devices.	
Smallwood and Thomas^57^	An inexpensive portable monitor for measuring evaporative water loss	2	Device is calibrated first with three different saturated salt solutions. Then used to test water loss rates from two subjects with C5/6 spinal lesions at seven different locations.	In vitro	Author fabricated evaporimeter	The monitor can measure evaporative water loss at normal rates of 10–20 g/m/h to an accuracy of about 10% of the reading.	
Steiner et al. ^27^	Side‐by‐side comparison of an open chamber (TM300) and a closed chamber (Vapometer) transepidermal water loss meter	17	Compared the results of TEWL measurements between two commonly used open and closed chamber TEWL devices. Five hundred and forty measurements were taken in 17 participants on the dorsum and palm of both hands on 2 days and the order of the devices was randomised. The TM300 was used in two modes: once as the standard open chamber model and second as a closed chamber model using the supplied semi‐permeable ring.	In vivo	Tewameter TM300 (Courage and Khazaka), Vapometer (Delphin Technologies)	The mean difference for open (TM300) minus closed method is 1.3 g/m^2^ h, with agreement limits of ‐5.4 and 8.2 g/m^2^ h. The agreement is very good for the range up to 20 g/m^2^ h. The measurements outside the agreement limit are predominantly for higher TEWL measurements. Comparing the Vapometer against the TM300 open chamber, the mean difference is 1.2 g/m^2^ h but the agreement limits are within the range of ‐35.8 and 38.3 g/m^2^ h difference. Both devices yield similar measurements in the range up to about 25 g/m^2^ h, and above an average of about 55 g/m^2^ h the TM300 records systematically lower readings compared with the Vapometer; this effect increases with increasing TEWL readings.	The intra‐class correlation coefficient (ICC) comparing the TM300 open chamber TEWL with the closed chamber version is 0.98 (95% CI: 0.97–0.98) and 0.70 (95% CI: 0.65–0.74) for the Vapometer.
Tagami et al.^53^	A portable device using a closed chamber system for measuring transepidermal water loss: comparison with the conventional method	21	Compared TEWL measurements between the H4300 and DermaLab evaporimeters in 21 healthy volunteers, four atopic dermatitis, three psoriatic patients aged 22—81 years. For healthy patients measurements were from cheek, flexor surface of forearm, extensor surface of leg. For patients with skin lesions measurements were taken from various lesions on the extremities. Measurements were at 50% humidity and 21°C.	In vivo	DermaLab (Cortex Technologies), H4300 (NIKKISO‐YSI Co, Ltd)	H4300 had variation of ∼15% when measured across different anatomical sites (higher on cheek than other areas). DermaLab 12.0 ± 1.0 cheek, 4.7 ± 0.5 forearm, 5.6 ± 0.7 leg (*p* < 0.0001). DermaLab CV 8.33, 10.64, 12.5%. Similar differences in closed chamber device: cheek (6.7 ± 0.6), forearm (2.3 ± 0.2), leg (2.8 ± 0.4) (*p *< 0.0001). Similarly results from patients with atopic dermatitis or psoriasis were higher than those from normal patients in both devices.	When all the data were compared, the TEWL values obtained with the closed system device correlated well with those obtained with the open system device, the DermaLab. An excellent linear relationship (*R* ^2^ = 0.92; *p* = 0.0001) was found, over a wide range of TEWL values, between the two different instruments
Van Sam et al.^43^	Transepidermal water loss management standardisation: kinetic and topographic aspects	34	TEWL measurements by the EP1C evaporimeter in 11 patients (aged 22—55 years) on three different sites (wrist, mid forearm, elbow). In the second set of experiments measurements were taken from seven patients from site 2 at 5 min intervals, for 2 h on 3 consecutive days to study reproducibility. Then measurements were taken from 16 patients on one day measured with the same intervals.	In vivo	Evaporimeter EP1 (Servomed AB)		ANOVA test showed no influence of subjects on variability in results and that inter‐subject reproducibility was good, and reproducibility was the same for the anatomical sties (*p* = 0.05). Hartley test showed that for each time, all patient values were the same and reproducibility was good.
Yamamura et al.^44^	Simple monochromatic refractometer for transepidermal water loss	44	Forty‐four patients (21 males, 23 females, mean age 42.61 years) with normal skin and 12 patients with various skin conditions. TEWL measurements from frontal surface of the lower leg at the same time by both devices.	In vivo	Noevir‐EVA, Evaporimeter EP1 (Servomed AB)		There was agreement between the Noevir‐EVA device and the commercially available Evaporimeter EP1 *r* = 0.984
Ye et al.^28^	Validation of GPSkin Barrier for assessing epidermal permeability barrier function and stratum corneum hydration in humans	200	Measurements in 200 patients (74 males, 126 females) aged 1–78 years. All patients had no soap, or detergent for 12 h and no skin care products for 24 h. All measurements were performed by Ye et al. TEWL and SC hydration were measured on the cheek, the dorsal hand, and the forearm (flexor site) with TM300 and Corneometer CM825, respectively, attached to a Courage and Khazaka MPA5 system. Readings were taken with a laptop connected to MPA5. When using GPSkin Barrier, perpendicularly placing the device on the measurement site for 10 s, both TEWL and SC hydration readings are shown on a smartphone.	In vivo	Tewameter TM300 (Courage and Khazaka), GPSkin Barrier Light (Gpower)	In the cheek TM300 mean ± SEM was (19.08 ± 0.77) and GPSkin (12.34 ± 0.65). Forearm: TM300 (17.8 ± 0.73) and GPSkin (10.87 ± 0.54). Dorsal hand: TM300 was (21.57 ± 0.8) and GPSkin was (14.96 ± 0.61).	Cheek *r* = 0.7009, *p* < 0.0001. In the forearm, *r* ^2^ = 0.6449, *p* < 0.0001. In the dorsal hand, *R* ^2^ = 0.6991, *p* < 0.0001.
Yoshihara et al.^29^	A new method of measuring the transepidermal water loss of dog skin	21	Study conducted on 19 beagles (11 males, 8 females, 1–5 years old) and two cross‐bred dogs (7 years old females). CC01 was compared to T300. TEWL measurements were also taken across different anatomical sites, different relative humidity's (58%, 78%) and different temperatures (18°C, 20°C, 22°C, 24°C, 26°C, 28°C). Hair was clipped on four different sites (lumbar back, shoulder, leg, tail base) were measure at each site five times with each device.	In vivo in dogs	Tewameter TM300 (Courage and Khazaka), TEWL analyser CC01	Results for T300 are as follows: back (mean = 17.78, variance 1.26, SD 1.12), leg (mean 23.22, variance 5.87, SD 2.42), tail base (mean 26.58, variance 8.48, SD 2.91) and shoulder (mean 65.82, variance 7.81, SD 2.79). For the CCO1 it was: back (mean 20.56, variance 1.05, SD 1.02), leg (mean 23.4, variance 1.77, SD 1.33), tail base (mean 26.76, variance 5.9, SD 2.43), shoulder (mean 40.19, mean 0.58, SD 0.76). The lower variance and SD indicates more reliable results with the CC01.	
Zhai et al.^35^	Tape stripping method in man: comparison of evaporimetric methods	10	Ten healthy patients (6 males, 4 females) had 10 or 20 strippings from bilateral forearms. Each patient/forearm was randomly allocated to have TEWL measurements using either an open or closed chamber device.	In vivo	Tewameter TM210 (Courage and Khazaka), Vapometer SWL‐3 (Delphin Technologies)	The closed chamber device showed a slightly higher (but not statistically significant) inter‐individual CV. Open chamber: 10 strips—mean TEWL 2.2 ± 0.9, CV (%) 41.7. 20 strips—TEWL 3.9 ±1.3, CV (%) 33.5. Closed chamber: 10 strips—2.4 ± 1.4, CV (%) 60.5%. 20 strips—TEWL 4.6 ± 2.2, CV (%) 47.8%.	There was no a statistically significant difference between two sites in measures by open and closed chamber devices, neither after 10 nor after 20 strips.

Abbreviations: AD, atopic dermatitis; ANOVA, analysis of variance; CI, confidence interval; CV, coefficient of variation; ICC, intra‐class correlation coefficient; LoA, limits of agreement; POSAS, The Patient and Observer Scar Assessment Scale; SC, stratum corneum; SD, standard deviation; SEM, standard error of measurement; SLS, sodium lauryl sulphate; TEWL, transepidermal water loss; THC, truncated hollow cone; VC, variation coefficient; WVTR, water vapour transmission rates.

## References

[1] Boer M , Duchnik E , Maleszka R , Marchlewicz M . Structural and biophysical characteristics of human skin in maintaining proper epidermal barrier function. Adv Dermatol Allergol. 2016;1:1–5. 10.5114/pdia.2015.48037 PMC4793052 26985171

[2] Merk HF . Barrier skin. In: Krutman J , Merk HF , editors. Environment and skin. Switzerland: Springer; 2018.

[3] Akdeniz M , Gabriel S , Lichterfeld‐Kottner A , Blume‐Peytavi U , Kottner J . Transepidermal water loss in healthy adults: a systematic review and meta‐analysis update. Br J Dermatol. 2018;179(5):1049–55. 30022486 10.1111/bjd.17025

[4] Berardesca E , Loden M , Serup J , Masson P , Rodrigues LM . The revised EEMCO guidance for the in vivo measurement of water in the skin. Skin Res Technol. 2018;24(3):351–8. 29923639 10.1111/srt.12599

[5] du Plessis J , Stefaniak A , Eloff F , John S , Agner T , Chou TC et al. International guidelines for the in vivo assessment of skin properties in non‐clinical settings: part 2. transepidermal water loss and skin hydration. Skin Res Technol. 2013;19(3):265–78. 23331328 10.1111/srt.12037 PMC4522909

[6] Lee KC , Dretzke J , Grover L , Logan A , Moiemen N . A systematic review of objective burn scar measurements. Burns Trauma. 2016;4:14. 27574684 10.1186/s41038-016-0036-x PMC4964074

[7] Fluhr JW , Feingold KR , Elias PM . Transepidermal water loss reflects permeability barrier status: validation in human and rodent in vivo and ex vivo models. Exp Dermatol. 2006;15(7):483–92. 16761956 10.1111/j.1600-0625.2006.00437.x

[8] Rosado C , Pinto P , Rodrigues LM . Comparative assessment of the performance of two generations of Tewameter: TM210 and TM300. Int J Cosmet Sci. 2005;24(4):237–41. 10.1111/j.1467-2494.2005.00270.x 18492192

[9] De Paepe K , Houben E , Adam R , Wiesemann F , Rogiers V . Validation of the VapoMeter, a closed unventilated chamber system to assess transepidermal water loss vs. the open chamber Tewameter. Skin Res Technol. 2005;11(1):61–9. 15691261 10.1111/j.1600-0846.2005.00101.x

[10] Anthonissen M , Daly D , Fieuws S , Massage P , Van Brussel M , Vranckx J et al. Measurement of elasticity and transepidermal water loss rate of burn scars with the Dermalab(R). Burns 2013;39(3):420–8. 23000371 10.1016/j.burns.2012.07.026

[11] Akdeniz M , Gabriel S , Lichterfeld‐Kottner A , Blume‐Peytavi U , Kottner J . Transepidermal water loss in healthy adults: a systematic review and meta‐analysis update. Br J Dermatol. 2018;179(5):1049–55. 30022486 10.1111/bjd.17025

[12] Stephenson M , Riitano D , Wilson S , Leonardi‐Bee J , Mabire C , Cooper K et al. Chapter 12: systematic reviews of measurement properties. In: JBI manual for evidence synthesis. JBI; 2020. Available from: https://synthesismanual.jbi.global

[13] Mokkink LB , Boers M , Vleuten CPMvd , Bouter LM , Alonso J , Patrick DL et al. COSMIN risk of bias tool to assess the quality of studies on reliability or measurement error of outcome measurement instruments: a Delphi study. BMC Med Res Methodol. 2020;20(1):293. 33267819 10.1186/s12874-020-01179-5 PMC7712525

[14] Klotz T , Maddern G , Caplash Y , Wagstaff M . Devices measuring transepidermal water loss of the skin: a systematic review protocol of measurement properties. JBI Evid Synth. 2021;19(10):2893–903. 34100828 10.11124/JBIES-20-00468

[15] Prinsen CAC , Mokkink LB , Bouter LM , Alonso J , Patrick DL , de Vet HCW et al. COSMIN guideline for systematic reviews of patient‐reported outcome measures. Qual Life Res. 2018;27(5):1147–57. 29435801 10.1007/s11136-018-1798-3 PMC5891568

[16] Nilsson GE . Measurement of water exchange through the skin. Med Biol Eng Compt. 1977;15:209–18. 10.1007/BF02441040 195148

[17] Alexander H , Brown S , Danby S , Flohr C . Research techniques made simple: transepidermal water loss measurement as a research tool. J Invest Dermatol. 2018;138(11):2295–300.e1. 30348333 10.1016/j.jid.2018.09.001

[18] Mokkink LB , Terwee CB , Patrick DL , Alonso J , Stratford PW , Knol DL et al. The COSMIN study reached international consensus on taxonomy, terminology, and definitions of measurement properties for health‐related patient‐reported outcomes. J Clin Epidemiol. 2010;63(7):737–45. 20494804 10.1016/j.jclinepi.2010.02.006

[19] Rosenkoetter U , Tate RL . Assessing features of psychometric assessment instruments: a comparison of the COSMIN checklist with other critical appraisal tools. Brain Impairment. 2017;19(1):103–18.

[20] Moher D , Liberati A , Tetzlaff J , Altman DG , Group P . Preferred reporting items for systematic reviews and meta‐analyses: the PRISMA statement. J Clin Epidemiol. 2009;62(10):1006–12. 19631508 10.1016/j.jclinepi.2009.06.005

[21] Gardien KL , Baas DC , de Vet HC , Middelkoop E . Transepidermal water loss measured with the Tewameter TM300 in burn scars. Burns 2016;42(7):1455–62. 27233677 10.1016/j.burns.2016.04.018

[22] Hon KL , Kung J , Leung T . Evaluating skin equipment for assessing childhood eczema. Aust J Dermatol. 2018;59:18. 10.1080/09546634.2018.1442551 29460656

[23] Hua W , Fan LM , Dai R , Luan M , Xie H , Li AQ et al. Comparison of two series of non‐invasive instruments used for the skin physiological properties measurements: the DermaLab(®) from Cortex Technology vs. the series of detectors from Courage & Khazaka. Skin Res Technol. 2017;23(1):70–8. 27637867 10.1111/srt.12303

[24] Kikuchi K , Asano M , Tagami H , Kato M , Aiba S . Comparison of the measuring efficacy of transepidermal water loss of a reasonably priced, portable closed‐chamber system device H4500 with that of rather expensive, conventional devices such as Tewameter(®) and Vapometer(®). Skin Res Technol. 2017;23(4):597–601. 28517733 10.1111/srt.12377

[25] Miteva M , Richter S , Elsner P , Fluhr JW . Approaches for optimizing the calibration standard of Tewameter TM 300. Exp Dermatol. 2006;15(11):904–12. 17002688 10.1111/j.1600-0625.2006.00482.x

[26] Rosado C , Pinto P , Rodrigues LM . Comparative assessment of the performance of two generations of Tewameter: TM210 and TM300. Int J Cosmet Sci. 2005;27(4):237–41. 18492192 10.1111/j.1467-2494.2005.00270.x

[27] Steiner M , Aikman‐Green S , Prescott GJ , Dick FD . Side‐by‐side comparison of an open‐chamber (TM 300) and a closed‐chamber (Vapometer™) transepidermal water loss meter. Skin Res Technol. 2011;17(3):366–72. 21492241 10.1111/j.1600-0846.2011.00509.x

[28] Ye L , Wang Z , Li Z , Lv C , Man MQ . Validation of GPSkin Barrier(®) for assessing epidermal permeability barrier function and stratum corneum hydration in humans. Skin Res Technol. 2019;25(1):25–9. 29863296 10.1111/srt.12590

[29] Yoshihara T , Shimada K , Momoi Y , Konno K , Iwasaki T . A new method of measuring the transepidermal water loss (TEWL) of dog skin. J Vet Med Sci. 2007;69(3):289–92. 17409646 10.1292/jvms.69.289

[30] Elkeeb R , Hui X , Chan H , Tian L , Maibach HI . Correlation of transepidermal water loss with skin barrier properties in vitro: comparison of three evaporimeters. Skin Res Technol. 2010;16(1):9–15. 20384878 10.1111/j.1600-0846.2009.00406.x

[31] Imhof RE , Xiao P , Berg EP , Ciortea LI . Rapid measurement of TEWL with a condenser‐chamber instrument. In: 15th International Meeting of the ISBS and 2nd Joint International Meeting of ISBS and ISSI, Philadelphia. 2005.

[32] Nuutinen J , Alanen E , Autio P , Lahtinen MR , Harvima I , Lahtinen T . A closed unventilated chamber for the measurement of transepidermal water loss. Skin Res Technol. 2003;9(2):85–9. 12709124 10.1034/j.1600-0846.2003.00025.x

[33] Farahmand S , Tien LL , Hui XY , Maibach HI . Measuring transepidermal water loss: a comparative in vivo study of condenser‐chamber, unventilated‐chamber and open‐chamber systems. Skin Res Technol. 2009;15(4):392–8. 19832948 10.1111/j.1600-0846.2009.00376.x

[34] Shah JH , Zhai H , Maibach HI . Comparative evaporimetry in man. Skin Res Technol. 2005;11(3):205–8. 15998333 10.1111/j.1600-0846.2005.00099.x

[35] Zhai H , Dika E , Goldovsky M , Maibach HI . Tape‐stripping method in man: comparison of evaporimetric methods. Skin Res Technol. 2007;13(2):207–10. 17374064 10.1111/j.1600-0846.2007.00218.x

[36] Lau‐Gillard PJ , Hill PB , Chesney CJ , Budleigh C , Immonen A . Evaluation of a hand‐held evaporimeter (VapoMeter) for the measurement of transepidermal water loss in healthy dogs. Vet Dermatol. 2010;21(2):136–45. 19961567 10.1111/j.1365-3164.2009.00738.x

[37] Blichmann CW , Serup J . Reproducibility and variability of transepidermal water loss measurement. Studies on the servo med evaporimeter. Acta Derm Venereol. 1987;67(3):206–10. 2442932

[38] Grove GL , Grove MJ , Zerweck C , Pierce E . Comparative metrology of the evaporimeter and the DermaLab® TEWL probe. Skin Res Technol. 1999;5(1):1–8.

[39] Park SJ , Tamura T . Measurement of regional evaporation rate from skin surface by evaporimeter. Ann Physiol Anthropol. 1992;11(4):417–23. 1388405 10.2114/ahs1983.11.417

[40] Pinnagoda J , Tupker RA , Coenraads PJ , Nater JP . Comparability and reproducibility of the results of water loss measurements: a study of 4 evaporimeters. Contact Derm. 1989;20(4):241–6. 10.1111/j.1600-0536.1989.tb03139.x 2752735

[41] Rogiers V . Transepidermal water loss measurements in patch test assessment: the need for standardisation. Curr Probl Dermatol. 1995;23:152–8. 9035908 10.1159/000424310

[42] Scott RC , Oliver GJ , Dugard PH , Singh HJ . A comparison of techniques for the measurement of transepidermal water loss. Arch Dermatol Res. 1982;274(1–2):57–64. 7165368 10.1007/BF00510358

[43] Van Sam V , Passet J , Maillols H , Guillot B , Guilhou JJ . TEWL measurement standardization: kinetic and topographic aspects. Acta Dermato‐Venereol. 1994;74(3):168–70. 10.2340/0001555574168170 7915454

[44] Yamamura T , Masaki H , Sakon K , Suzuki K , Tezuka T . Simple monochromatic refractometer for trans‐epidermal water loss (TEWL). J Dermatol Sci. 1990;1(3):201–6. 2085507 10.1016/0923-1811(90)90132-w

[45] Grinich EE , Shah AV , Simpson EL . Validation of a novel smartphone application‐enabled, patient‐operated skin barrier device. Skin Res Technol. 2019;25(5):612–7. 30942506 10.1111/srt.12692

[46] Grinich EE , Topham C , Haynes D , Chung J , Latour E , Simpson EL . Validation of a novel patient‐operated device for measuring skin barrier function in atopic dermatitis. Skin Res Technol. 2021;27(5):824–30. 33665909 10.1111/srt.13027

[47] Logger JGM , Driessen RJB , de Jong E , van Erp PEJ . Value of GPSkin for the measurement of skin barrier impairment and for monitoring of rosacea treatment in daily practice. Skin Res Technol. 2021;27(1):15–23. 10.1111/srt.12900 PMC7984125 32573826

[48] Murphrey MB , Erickson T , Canter T , Rangel SM , Paller AS . Can a handheld device accurately measure barrier function in ichthyoses? Pediatr Dermatol. 2020;37(5):860–3. 32748517 10.1111/pde.14305

[49] Cointereau‐Chardon S , Caberlotto E , Vicic M , Flament F . Self‐recording the skin hydration and trans‐epidermal water loss parameters: a pilot study. Skin Res Technol. 2020;26(5):713–7. 32227383 10.1111/srt.12862

[50] Sim D , Kim SM , Kim SS , Doh I . Portable skin analyzers with simultaneous measurements of transepidermal water loss, skin conductance and skin hardness. Sensors 2019;19(18):3857. 31500135 10.3390/s19183857 PMC6767198

[51] Anthonissen M , Daly D , Fieuws S , Massagé P , Van Brussel M , Vranckx J et al. Measurement of elasticity and transepidermal water loss rate of burn scars with the Dermalab(®). Burns 2013;39(3):420–8. 23000371 10.1016/j.burns.2012.07.026

[52] Fell M , Meirte J , Anthonissen M , Maertens K , Pleat J , Moortgat P . The Scarbase Duo®: intra‐rater and inter‐rater reliability and validity of a compact dual scar assessment tool. Burns 2016;42(2):336–44. 26774602 10.1016/j.burns.2015.08.005

[53] Tagami H , Kobayashi H , Kikuchi K . A portable device using a closed chamber system for measuring transepidermal water loss: comparison with the conventional method. Skin Res Technol. 2002;8(1):7–12. 12005122

[54] Peer RP , Burli A , Maibach HI . Unbearable transepidermal water loss (TEWL) experimental variability: why? Arch Dermatol Res. 2022;314(2):99–119. 33638033 10.1007/s00403-021-02198-y

[55] Barel AO , Clarys P . Study of the stratum corneum barrier function by transepidermal water loss measurements: comparison between two commercial instruments: evaporimeter and tewameter. Skin Pharmacol. 1995;8(4):186–95. 7488395 10.1159/000211345

[56] Norlén L , Engblom J , Andersson M , Forslind B . A new computer‐based evaporimeter system for rapid and precise measurements of water diffusion through stratum corneum in vitro. J Invest Dermatol. 1999;113(4):533–40. 10504437 10.1046/j.1523-1747.1999.00727.x

[57] Smallwood RH , Thomas SE . An inexpensive portable monitor for measuring evaporative water loss. Clin Phys Physiol Meas. 1985;6(2):147–54. 4017445 10.1088/0143-0815/6/2/006

[58] Bak M , Tarapata G , Weremczuk J . Portable device, based on a microcontroller, for measurement of TEWL factor. In: Romaniuk RS , editor. Photonics applications in astronomy, communications, industry, and high‐energy physics experiments, Pts 1 and 2. SPIE; 2006.

[59] Baker H , Kligman AM . Measurement of transepidermal water loss by electrical hygrometry. Instrumentation and responses to physical and chemical insults. Arch Dermatol. 1967;96(4):441–52. 6046392

[60] Cohen JC , Hartman DG , Garofalo MJ , Basehoar A , Raynor B , Ashbrenner E et al. Comparison of closed chamber and open chamber evaporimetry. Skin Res Technol. 2009;15(1):51–4. 19152579 10.1111/j.1600-0846.2008.00334.x

[61] Imhof RE , De Jesus ME , Xiao P , Ciortea LI , Berg EP . Closed‐chamber transepidermal water loss measurement: microclimate, calibration and performance. Int J Cosmet Sci. 2009;31(2):97–118. 19175433 10.1111/j.1468-2494.2008.00476.x

[62] Petro AJ , Komor JA . Correction to absolute values of evaporation rates measured by the Servo‐Med® evaporimeter. Bioeng Skin. 1987;3(3):271–80.

[63] Valentin B , Mundlein M , Chabicovsky R , Nicolics J . A novel transepidermal water loss sensor. IEEE Sens J. 2006;6(4):1022–6.

[64] Weigmann HJ , Ulrich J , Schanzer S , Jacobi U , Schaefer H , Sterry W et al. Comparison of transepidermal water loss and spectroscopic absorbance to quantify changes of the stratum corneum after tape stripping. Skin Pharmacol Physiol. 2005;18(4):180–5. 15908758 10.1159/000085863

